# WHO International Standards for antibodies to HPV6 HPV11 HPV31 HPV33 HPV45 HPV52 and HPV58

**DOI:** 10.1038/s41541-024-00949-2

**Published:** 2024-09-10

**Authors:** Troy J. Kemp, Gitika Panicker, Carina Eklund, Jianhui Nie, Youchun Wang, Simon Beddows, Peter Rigsby, Weijin Huang, Joakim Dillner, Elizabeth R. Unger, Ligia A. Pinto, Dianna E. Wilkinson, the collaborative study participants, Paul Licciardi, Zheng Quan Toh, Martin Müller, T. M. Chozhavel Rajanathan, Shaowei Li, Ningshao Xia, Ge Liu, Chenliang Zhou, Lingyun Zhou, Nan Xu, Kavita Panwar, Denise Galloway, Jody Carter

**Affiliations:** 1 Vaccine, Immunity, and Cancer Directorate, Frederick National Laboratory for Cancer Research, Frederick, MD USA; 2 Centers for Disease Control and Prevention, National Center for Emerging and Zoonotic Infectious Diseases, Division of High-Consequence Pathogens and Pathology, Atlanta, GA USA; 3 Karolinska Institutet, Karolinska Universitetssjukhuset, Huddinge, Sweden; 4 National Institutes for Food and Drug Control, Beijing, PR China; 5 UK Health Security Agency, Virus Reference Department, London, UK; 6 Medicines and Healthcare products Regulatory Agency (MHRA), South Mimms, UK; 7 Murdoch Children’s Research Institute, New Vaccines Group, Melbourne, Australia; 8 German Cancer Research Center, Heidelberg, Germany; 9 Cadila Healthcare Limited, Vaccine Technology Center, Ahmedabad, Gujarat India; 10 National Institute of Diagnostics and Vaccine Development in Infectious Diseases, Xiamen University, Xiamen, P. R. China; 11 Shanghai Zerun Biotech Co., Ltd, Shanghai, PR China; 12 Fred Hutchinson Cancer Research Center, Seattle, Washington USA

**Keywords:** Predictive markers, Cervical cancer

## Abstract

Previously established World Health Organization (WHO) International Standards (IS) for anti-HPV16 and HPV18 antibodies are used to harmonize results across human papillomavirus (HPV) serology assays. Here, we present an international collaborative study to establish ISs for antibodies against HPV6 (NIBSC code 19/298), HPV11 (20/174), HPV31 (20/176), HPV33 (19/290), HPV45 (20/178), HPV52 (19/296) and HPV58 (19/300). The candidate standards were prepared using sera from naturally infected individuals. Each candidate was shown to be monospecific for reactivity against its indicated HPV type except for the HPV11 candidate, which was also reactive against other types. Expression of antibody levels relative to the relevant candidate IS reduced inter-laboratory variation allowing greater comparability between laboratories. Based on these results, the WHO Expert Committee on Biological Standardization established each of the 7 candidates as the 1st IS for antiserum to its indicated HPV type for use in the standardization of HPV pseudovirion-based neutralization and antibody-binding assays.

## Introduction

In August 2020 the World Health Assembly adopted the global strategy for cervical cancer elimination^1,2^. Vaccination against human papillomavirus (HPV) and surveillance play crucial roles in this initiative^1^. Accurate and reproducible HPV serology assays are essential for assessing the immunogenicity of HPV vaccines, as well as monitoring vaccine quality and performance in different populations^3,4^. HPV serology standardization is also critical for measuring antibody responses from past or present HPV infections in epidemiological studies, for example, monitoring the spread of HPV infections via antibody responses in different populations – a key feature for both planning of optimal HPV control programs and to follow up on their success^5^.

A World Health Organization (WHO) collaborative pilot study conducted in 2005 demonstrated that the availability of WHO International Standards (ISs) for antibodies to HPV would facilitate the standardization of HPV serological methods^5^. In the absence of such standards, individual laboratories apply their own reference samples to standardize assays within the laboratory. However, such in-house standards are not usually harmonized with other laboratories and methods, and thus cannot serve to improve the reproducibility and comparability between laboratories.

The WHO’s Expert Committee on Biological Standardization (ECBS) establishes reference standards for biological substances used in the prevention, treatment, or diagnosis of human disease^6,7^. International Standards are recognized as the highest-order references for biological substances and are assigned potencies in arbitrary International Units (IU)^6^. Their primary purpose is to calibrate secondary reference standards in terms of the IU for use in laboratory assays, thus providing a globally recognized results-reporting system that allows traceability of measurements across studies independent of the methods used^6,8,9^.

**Fig. 1:**
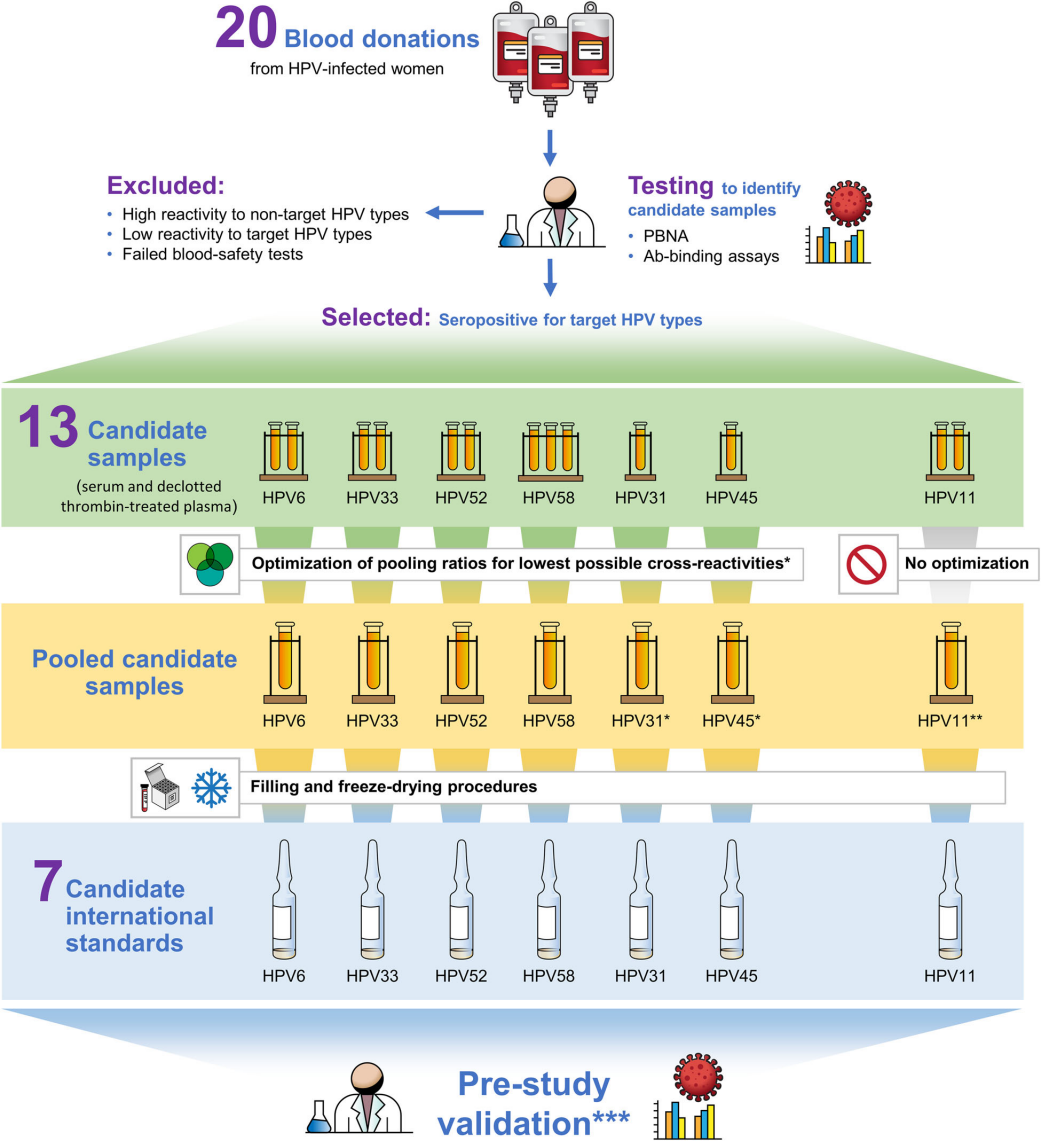
Process flow diagram for testing, selection and formulation of donations from naturally infected women to produce the 7 candidate WHO International Standards for HPV antibodies. Twenty anonymized donations obtained from women naturally infected with Human Papillomavirus (HPV) were provided for initial testing in HPV type-specific pseudovirion-based neutralization assays (PBNA) and antibody binding (Ab-binding) assays. Thirteen candidate samples shown to be seropositive for the target HPV types were selected for further development. Candidate samples for antibodies to HPV6, HPV31, HPV33, HPV45, HPV52 and HPV58 were selected for optimization of pooling ratios to obtain lowest possible cross-reactivities for non-target HPV types. The HPV11 candidate samples were pooled without optimization. The candidate samples were then filled into ampoules and freeze-dried in separate manufacturing procedures to produce the 7 candidate International Standards. Prior to their formal assessment in the multicenter international collaborative study, the candidate International Standards underwent validation testing in PBNA and Ab-Binding assays. The single asterisk indicates that seronegative serum was used for optimizing the reactivities of candidate samples for HPV31 and HPV45 antibodies. The double asterisk indicates that pooling of candidate samples for HPV11 antibodies was not optimized based on the exception criteria of “no type-cross-reactivity” due to difficulty in sourcing monospecific material. The triple asterisk indicates that the candidate International Standards for HPV31 and HPV45 antibodies were validated after the optimization procedure. The candidate International Standards for HPV6, HPV11, HPV33, HPV52 and HPV58 were validated after freeze-drying.

To assure the quality and efficacy of HPV virus-like particle (VLP) vaccines, WHO recommends that antibody levels should be reported in IU for HPV types for which an IS is available^3^. International Standards for antibodies against high-risk HPV16 and HPV18 were established by WHO ECBS in 2009 and 2012, respectively^10–12^, and a proposal for the development and establishment of ISs for antibodies against low-risk HPV6 and HPV11 and high-risk HPV31, HPV33, HPV45, HPV52 and HPV58 was endorsed by ECBS in October 2016 through collaborative efforts led by NIBSC and the Frederick National Laboratory for Cancer Research^13^. Like the previously established HPV16 and HPV18 ISs, these would be derived from antisera obtained from women naturally infected with a single HPV type and produced according to WHO guidelines^6^ (Fig. 1).

A multicenter international collaborative study was then conducted across 11 laboratories to evaluate the suitability of the candidate standards to serve as 1^st^ WHO ISs for antibodies to HPV6, 11, 31, 33, 45, 52 and 58. Antibody responses of the candidates were evaluated using an array of HPV type-specific pseudovirion (PsV)-based neutralization assays (PBNA) and antibody binding (Ab-binding) assays. The aims of this collaborative study were to:

- assess the suitability of each candidate to serve as the IS for antibodies to its specified HPV type (HPV6, 11, 31, 33, 45, 52, or 58) and assign each a unitage in IU per ampoule for use in the calibration of PBNA and Ab-binding assays,
- characterize each candidate IS in terms of reactivity and specificity,
- assess each candidate’s potency i.e., level of specific reactivity in a range of typical assays performed in different laboratories,
- assess commutability i.e., establish the extent to which the candidates are suitable to serve as ISs for the variety of different samples being assayed, including samples from recipients of HPV vaccines, naturally infected individuals, and seronegative samples^6^.

The collaborative study findings presented here led to the establishment of the HPV International Standards for 7 HPV types contained in the currently licensed HPV vaccines. This is a remarkable advance for the HPV field enabling, for the first time, comparison of antibody responses between different studies.

## Results

### Data collection

HPV serology laboratories tested the samples using their established methods. Data on 17 samples (Table 1) were sent to the National Institute for Biological Standards and Control (NIBSC) by 11 laboratories. Participating laboratory affiliations included government research, public health, medical research, and regulatory organizations as well as HPV vaccine developers and manufacturers. Nine laboratories (Lab-3, 4, 5, 6, 7, 8, 9, 10, and 11) returned data for PBNA and 6 laboratories (Lab-1, 2, 6, 7, 8, and 10) for Ab-binding assays (Supplementary Table 1). With 2 exceptions, laboratories tested samples for antibodies against HPV6, 11, 16, 18, 31, 33, 45, 52, and 58 which are included in currently approved vaccines (hereinafter referred to collectively as the 9 HPV types). Lab-2 tested the samples for HPV6, 11, 16 and 18 in Ab-binding assays. Lab-9 returned data for samples in PBNA for antibodies against HPV18 and HPV45. In all, the laboratories returned 124 data sets covering the 9 HPV types with at least 3 independent assay runs performed per set.

**Table 1:** Samples included in the collaborative study

Blinded sample code	Description	Expected reactivity
X	Frozen pooled sera from recipients of 9-valent (9v) vaccine (9v vaccinee reference). Participants were instructed to include Sample X on every plate for a given assay	Validated high titers for antibodies to HPV types 6, 11, 16, 18, 31, 33, 45, 52 and 58 Will be described elsewhere.
A	NIBSC 19/290; anti-HPV33 IS candidate (duplicate of J)	Reactive to HPV33 and no other HPV vaccine type in validation assays
B	NIBSC 19/296; anti-HPV52 IS candidate (duplicate of K)	Reactive to HPV52 and no other HPV vaccine type in validation assays
C	Frozen serum from naturally infected woman (anonymous donor W520628)	Reactive to HPV33 across screening assays. Inconsistent reactivity to other HPV vaccine types
D	Frozen serum from naturally infected woman (anonymous donor S520160905)	Reactive to HPV52 in screening PBNA. Inconsistent reactivity to HPV52 in antibody-binding assays
E	Frozen pooled sera negative for antibodies to HPV vaccine types. This material was used in the formulation of HPV31 and HPV45 IS candidates	Non-reactive to HPV vaccine types
F	NIBSC 19/298; anti-HPV6 IS candidate	Reactive to HPV6 and no other HPV vaccine type in validation assays
G	NIBSC 20/174; anti-HPV11 IS candidate	Reactive to HPV11. Also reactive to HPV6 and HPV33 in some validation assays
H	Frozen serum from naturally infected woman (component of F)	Reactive to HPV6 and no other HPV vaccine type
I	Frozen pooled plasma from recipients of bi-valent vaccine plasma (2v vaccinee reference). (duplicate of P)	High titers for antibodies to HPV16 and HPV18. Intermediate titers to non-vaccine HPV31 and HPV45. Low levels of antibodies generated by vaccination and/or natural infection toward other α and β HPV types cannot be ruled out^14^
J	NIBSC 19/290; anti-HPV33 IS candidate (duplicate of A)	Reactive to HPV33 and no other HPV vaccine type in validation assays
K	NIBSC 19/296; anti-HPV52 IS candidate (duplicate of B)	Reactive to HPV52 and no other HPV vaccine type in validation assays
L	NIBSC 19/300; anti-HPV58 IS candidate	Reactive to HPV58 and no other HPV vaccine type in validation assays
M	Frozen serum from woman non-reactive to vaccine types in initial screening [01159].	Reactive for HPV68 antibodies. Initially considered sero-negative for other HPV types but low-level reactivity observed for HPV16 and HPV58 in validation PBNA
N	NIBSC 20/176; anti-HPV31 IS candidate	Monospecific for HPV31 reactivity in mixing study
O	NIBSC 20/178; anti-HPV45 IS candidate	Monospecific for HPV45 reactivity in mixing study
P	Frozen pooled plasma from recipients of bi-valent vaccine plasma (2v vaccinee reference). (duplicate of I)	High titers for antibodies to HPV16 and HPV18. Intermediate titers to non-vaccine HPV31 and HPV45. Low levels of antibodies generated by vaccination and/or natural infection toward other α and β HPV types cannot be ruled out^14^

### Intra-assay and inter-assay variability of laboratory estimates of antibody concentrations

**Fig. 2:**
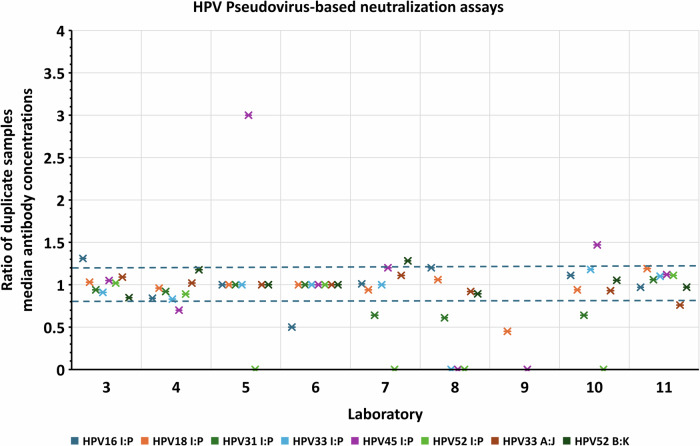
Assessment of intra-assay variability of duplicate samples tested in PBNA. Ratios of median absolute antibody concentrations (un-transformed) plotted for duplicate samples (I:P, A:J and B:K) tested for HPV16, 18, 31, 33, 45 and 52 antibodies. A ratio of 1 indicates that the duplicates had matching results. A greater than 20% difference in results for duplicate samples was selected as a guideline to identify greater variability (blue dashed lines mark the 0.8 and 1.2 ratio cut-offs). Data points plotted as 0 on the x axis indicates at that 1 sample (I or P) was reported below the assay cut-off. Ratios of potencies for HPV6, HPV11 and HPV58 for duplicates I and P were not determined as ≥ 50% of laboratories scored the samples negative.

**Fig. 3:**
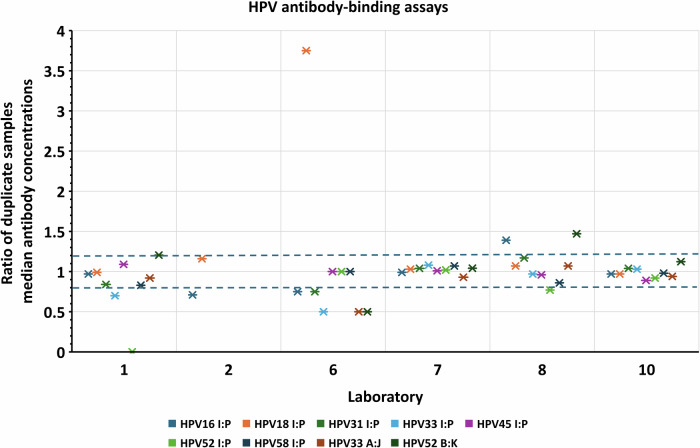
Assessment of intra-assay variability of duplicate samples tested in Ab-binding assays. Ratios of median absolute antibody concentrations (un-transformed) plotted for duplicate samples (I:P, A:J and B:K) tested for HPV16, 18, 31, 33, 45, 52 and 58 antibodies. A ratio of 1 indicates that the duplicates had matching results. A greater than 20% difference in results for duplicate samples was selected as a guideline to identify greater variability (blue dashed lines mark the 0.8 and 1.2 ratio cut-offs). Data points plotted as 0 on the x-axis indicates at that 1 sample (I or P) was reported below the assay cut-off. Ratios for responses to HPV6 and HPV11 for duplicates I and P were not determined as ≥ 50% of laboratories scored the samples negative.

Within-assay agreement for duplicate samples (I & P, J & A, K & B) indicates the extent of intra-assay variation of median antibody concentrations. Most laboratory estimates for duplicate samples were within a difference of 20% (ratios ranged from 0.8 to 1.20) indicating acceptable intra-assay variability (Figs. 2 and 3; Supplementary Table 2).

**Fig. 4:**
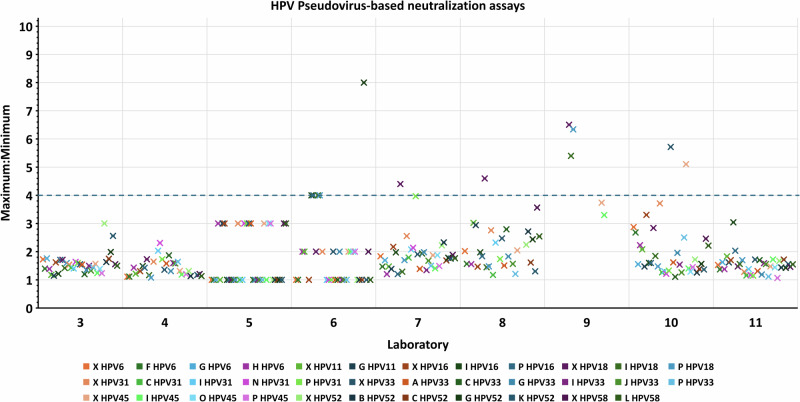
Assessment of inter-assay (within-laboratory) variability for samples tested in PBNA. Ratios of maximum and minimum laboratory estimates of HPV antibody concentration (un-transformed) for samples tested in at least 3 independent PBNA. A greater than 4-fold difference (blue dashed line cut-off) in sample antibody concentrations across assays was selected as a guideline to identify greater inter-assay variability.

**Fig. 5:**
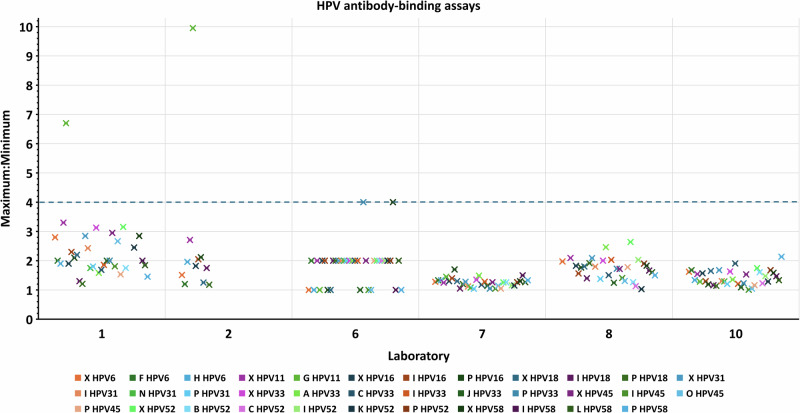
Assessment of inter-assay (within-laboratory) variability for samples tested in Ab-binding assays. Ratios of maximum and minimum laboratory estimates of HPV antibody concentration (un-transformed) for samples tested in at least 3 independent Ab-binding assays. A greater than 4-fold difference (blue dashed line cut-off) in sample antibody concentrations across assays was selected as a guideline to identify greater inter-assay variability.

Agreement for samples across independent assays within a laboratory indicates the extent of inter-assay variation of antibody concentrations. In nearly all cases (97.0% of PBNA and 98.9% of Ab-binding), the spreads of maximum and minimum (Max:Min) estimates of antibody concentration across assays were no more than 4-fold, indicating acceptable within-laboratory variability (Figs. 4, 5).

### Detection of HPV antibodies in PBNA and Ab-binding assays

The positive/negative scorings for detection of antibodies based on laboratory-defined cut-offs for the 9 HPV types are listed in Supplementary Tables 3–11 along with laboratory median estimated antibody concentrations and relative potencies for samples scored overall positive. The starting dilution used by each laboratory differed, but negative serum sample E was scored negative for the 9 types by most laboratories except in 3 cases, where sample E scored positive near the laboratory-defined PBNA cut-off for detection (Lab-3, Lab-11 for anti-HPV6, Supplementary Table 3; Lab-3 for anti-HPV52, Supplementary Table 10).

As expected for sera/plasma obtained from vaccinated individuals, sample X (9-valent [9v] vaccinated reference) and duplicate samples I & P (2-valent [2v] vaccinated reference) were scored positive by all laboratories for antibodies to vaccine types HPV16 and HPV18. The laboratories also scored sample X seropositive for vaccine types HPV6, 11, 31, 33, 45, 52, 58; and samples I & P seropositive for non-vaccine HPV31, 33 and 45 (except for Lab-8/PBNA which scored sample I negative for HPV33 and 45 antibodies and sample P negative for HPV45 antibodies). Samples I & P were scored negative for antibodies to non-vaccine HPV6 and 11 by ≥ 50% laboratories. The scorings for samples I & P for antibodies to non-vaccine HPV52 and 58 were mixed with ≥ 75% Ab-binding compared to < 50% PBNA results scoring the samples seropositive for both types. Whether induced by vaccination or natural infection, any cross-reactivity observed for the 2v vaccinated reference against non-vaccine HPV types is expected to be intermediate or low compared to antibody levels of vaccine HPV types^14^.

The specificity of each candidate standard was assessed using the positive/negative scorings for reactivity against the 9 HPV types (Supplementary Table 12). The candidates with good laboratory agreement for specificity to their respective target genotype were samples F (HPV6), N (HPV31), duplicates A & J (HPV33), O (HPV45), duplicates B & K (HPV52), and L (HPV58). The HPV11 candidate standard (Sample G) was reactive to HPV11 in 6 out of 8 laboratories for PBNA and 3 of 4 laboratories for Ab-binding assays. Sample G also had reactivity against HPV6, 33, 52 and 58 in some assays (predominantly PBNA). This cross-reactivity is not unexpected based on prior validation results (see Methods).

### Absolute antibody concentrations and assessment of variability across laboratories

Consistent with vaccine-induced antibody responses to vaccine types, the geometric means (GM) of absolute antibody concentrations of the 9v-vaccinated reference for HPV6, 11, 31, 33, 45, 52, and 58 were orders of magnitude higher than those observed for the candidate standards (which are from naturally infected individuals) (PBNA, Table 2; Ab-binding, Table 3. See Supplementary Tables 3–11 for individual laboratory median estimated antibody concentrations and relative potencies). Also, the GM antibody concentrations of natural infection samples were more akin to those of the candidate standards than to those of the 9v vaccinated reference. The 2v vaccinated reference had GM antibodies concentrations for non-2v-vaccine types HPV31 & 45 that were intermediate to responses observed for natural infection versus vaccine types. GM responses observed for the 2v reference non-vaccine HPV52 & HPV58 in Ab-binding assays were within 1 order of magnitude of the respective candidate standard.

**Table 2:** Summary of PBNA results across laboratories

PBNA	Sample	n/N*	Overall GM titer	%GCV	Max:Min	n/N	Overall GM relative potency	%GCV	Max:Min
Antibody									
HPV6	F-Candidate standard	8/8	274	126	12.9	8/8	1	0	1
	X-Pooled 9v vaccinee serum	8/8	9437	217	35.9	8/8	34.40	93	5.9
	H- Natural infection	8/8	112	121	12.1	8/8	0.41	76	5.4
	G- Natural infection**	5/8	88	137	8.9	5/8	0.23	53	3.0
HPV11	G-Candidate standard**	6/8	245	203	19.3	6/8	1	0	1
	X-Pooled 9v vaccinee serum	8/8	11673	348	78.2	6/8	83.32	54	3.4
HPV31	N-Candidate standard	7/8	113	63	3.8	7/8	1	0	1
	X-Pooled 9v vaccinee serum	8/8	6810	92	7.3	7/8	60.31	144	10.1
	I- Pooled 2v vaccinee plasma duplicate	8/8	518	93	5.9	7/8	5.19	51	3.2
	P- Pooled 2v vaccinee plasma duplicate	8/8	624	80	4.0	7/8	5.98	35	2.3
	C- natural infection	6/8	73	57	3.2	5/8	0.75	29	2.0
HPV33	A-Candidate standard duplicate	8/8	312	106	7.3	8/8	1	0	1
	J-Candidate standard	8/8	304	92	5.6	8/8	0.97	13	1.5
	X-Pooled 9v vaccinee serum	8/8	22715	115	14.6	8/8	72.71	45	2.8
	I- Pooled 2v vaccinee plasma duplicate	7/8	92	137	10.3	7/8	0.26	60	4.5
	P- Pooled 2v vaccinee plasma duplicate	8/8	86	124	9.4	8/8	0.28	61	4.5
	C-Natural infection	8/8	674	104	7.6	8/8	2.16	48	4.0
	G- Natural infection**	8/8	158	71	4.1	8/8	0.51	53	3.4
HPV45	O-Candidate standard	6/9	96	99	6.6	6/9	1	0	1
	X-Pooled 9v vaccinee serum	9/9	6983	149	19.4	6/9	71.31	122	10.1
	I- Pooled 2v vaccinee plasma duplicate	8/9	111	114	10.9	6/9	1.39	69	4.4
	P- Pooled 2v vaccinee plasma duplicate	7/9	109	93	4.8	6/9	1.03	52	3.1
HPV52	B-Candidate standard	8/8	476	97	9.2	8/8	1	0	1
	K-Candidate standard	8/8	466	102	10.3	8/8	0.98	15	1.5
	X-Pooled 9v vaccinee serum	8/8	7831	126	11.7	8/8	16.46	63	4.8
	C-Natural infection	7/8	142	48	3.1	7/8	0.25	33	2.0
	D natural infection	7/8	132	117	6.1	7/8	0.28	106	7.7
	G natural infection	7/8	56	68	5.3	7/8	0.10	76	6.5
HPV58	L-Candidate standard	8/8	803	120	6.7	8/8	1	0	1
	X-Pooled 9v vaccinee serum	8/8	9924	112	8.8	8/8	12.35	103	7.3

The overall geometric mean (GM) of absolute titers for samples tested in PBNA are listed along with geometric coefficients of variation (%GCV) and ratios of maximum and minimum titers (Max:Min) which are measures of inter-laboratory variability. Also shown are GM potencies of samples expressed relative to the relevant candidate standard with corresponding %GCV and Max:Min. For the purpose of this assessment, candidate standards were assigned a value of 1 to determine overall GM relative potencies. Median titers across independent assays for each laboratory and HPV type are listed in Supplementary Tables 3–11. *n/N number of median responses/number laboratories testing. **Sample G is the candidate standard for HPV11 antibodies and is also reactive against other types in some assays.

**Table 3:** Summary of Ab-binding results across laboratories

Ab-binding	Sample	n/N*	Overall GM response	%GCV	Max:Min	n/N	Overall GM relative potency	%GCV	Max:Min
Antibody									
HPV6	F-Candidate standard	5/6	67	3596	1684	5/6	1	0	1
	X-Pooled 9v vaccinee serum	6/6	904	1087	266	5/6	20.35	219	16.2
	H- Natural infection	5/6	58	3923	2286	5/6	0.87	12	1.4
HPV11	G-Candidate standard	5/6	40	3334	2286	5/6	1	0	1
	X-Pooled 9v vaccinee serum	6/6	1825	1500	552	5/6	63.18	88	5.1
HPV31	N-Candidate standard	4/5	22	1409	667	4/5	1	0	1
	X-Pooled 9v vaccinee serum	4/5	900	758	267	4/5	52.68	54	2.5
	I- Pooled 2v vaccinee plasma duplicate	5/5	197	1142	750	4/5	11.50	34	2.0
	P- Pooled 2v vaccinee plasma duplicate	5/5	206	1268	842	4/5	12.68	54	2.7
HPV33	A-Candidate standard	5/5	21	951	615	5/5	1	0	1
	J-Candidate standard	5/5	18	722	333	5/5	0.85	35	2.1
	X-Pooled 9v vaccinee serum	5/5	1308	932	538	5/5	60.96	71	3.3
	I- Pooled 2v vaccinee plasma duplicate	5/5	33	2591	9142	5/5	1.54	183	14.9
	P- Pooled 2v vaccinee plasma duplicate	5/5	40	3136	12800	5/5	1.88	254	20.8
	C-Natural infection	5/5	69	838	513	5/5	3.20	42	2.4
HPV45	O-Candidate standard	5/5	24	846	421	5/5	1	0	1
	X-Pooled 9v vaccinee serum	5/5	580	606	115	5/5	24.67	86	4.9
	I- Pooled 2v vaccinee plasma duplicate	5/5	190	1081	366	5/5	8.06	117	6.6
	P- Pooled 2v vaccinee plasma duplicate	5/5	192	1077	400	5/5	8.14	104	5.4
HPV52	B-Candidate standard	5/5	48	822	303	5/5	1	0	1
	K-Candidate standard	5/5	48	1264	727	5/5	1.00	50	2.9
	X-Pooled 9v vaccinee serum	5/5	852	762	268	5/5	17.64	16	1.5
	I- Pooled 2v vaccinee plasma duplicate	4/5	70	2242	1157	4/5	0.88	157	8.3
	P- Pooled 2v vaccinee plasma duplicate	4/5	76	2058	896	4/5	0.96	136	6.5
	C natural infection	3/5	19	2403	268	3/5	0.22	81	3.3
HPV58	L-Candidate standard	5/5	36	1276	1333	5/5	1	0	1
	X-Pooled 9v vaccinee serum	5/5	1209	816	400	5/5	33.75	82	4.5
	I- Pooled 2v vaccinee plasma duplicate	5/5	34	2403	6400	5/5	0.94	152	12.6
	P- Pooled 2v vaccinee plasma duplicate	5/5	36	2257	5333	5/5	1.00	148	12.3

The overall geometric mean (GM) of absolute responses for samples tested in Ab-binding assays are listed with corresponding geometric coefficients of variation (%GCV) and maximum-to-minimum ratios (Max:Min) which are measures of inter-laboratory variability. Also shown are GM potencies of samples expressed relative to the relevant candidate standard with corresponding %GCV and Max:Min. For the purpose of this assessment, candidate standards were assigned a value of 1 to determine overall GM relative potencies. Median responses across independent assays for each laboratory and HPV type are listed in Supplementary Tables 3–11. *n Positive scores plus titers reported by Lab-2 and Lab-6. *n/N Number of median responses/number laboratories testing.

There was considerable variation in absolute antibody concentrations across laboratories. For PBNA, the differences between the maximum and minimum titers (Max:Min) ranged from 3.1 to 78.2-fold. Roughly half of the GM titers had inter-laboratory geometric coefficients of variation (%GCV)s between 48 and 348% (Table 2). The inter-laboratory variability was much greater for Ab-binding assays where %GCVs ranged from 606 to 3923% and Max:Min differences ranged from 115 to 12800-fold reflecting the use of laboratory-defined units which are not equivalent and making it impossible to compare results across laboratories (Table 3).

### Harmonization of PBNA and Ab-binding results by the candidate International Standards

The variability of results between laboratories was reduced for both PBNA and Ab-binding assays when relative potencies were used (Tables 2 and 3). The improvement in laboratory agreement was most pronounced for the Ab-binding assays where, before harmonization, none of the GM antibody concentrations had GCVs below 606%. By reporting the results relative to the relevant candidate standard, the comparability of Ab-binding results across laboratories was possible, with most sample GM potencies having inter-laboratory %GCVs less than 100% (and all less than 254%). The Max:Min spread of Ab-binding potencies ranged from 1.4 to 20.8-fold, which is also an improvement upon absolute results. Harmonization of PBNA results across laboratories was also seen when reported as relative potencies with %GCVs ranging from 13% to 144% and Max:Min differences of 1.5 to 10.1-fold.

As seen for the absolute results discussed above, the 9v vaccinated reference sample had higher antibody potencies than the candidate standards and other naturally immune sera as well as the 2v reference for non-vaccine types.

There were observed differences between the assay methods in HPV31, 33, 45 relative potencies for the 2v vaccinated reference, with the Ab-binding assays tending to give higher relative potencies compared to PBNA (Tables 2 and 3).

Some differences between the assay methods were noted for relative potencies for natural infection sample G in HPV6 and 52 assays, sample C in HPV31 assays, and Sample D in HPV52 assays (Tables 2 and 3, Supplementary Tables 3, 7 and 10). In these cases, the Ab-binding assays tended to be negative or low-positive while some or most of the PBNAs were positive. The equivocal results across laboratories for some samples may be due to the assays operating at their limits of sensitivity.

### Stability study

The long-term stabilities of the candidate standards were assessed through accelerated thermal degradation (ATD) studies, which allow the prediction of degradation rates for samples stored at low temperatures (e.g. −20 °C) based on the observed loss in potency of samples stored at elevated temperatures (e.g., 4 °C, 20 °C, 37 °C, 45 °C)^15^. The potencies of the ATD samples tested in PBNA by reference laboratories REF-1 and REF-2 (Supplementary Table 13) show that there was little to no loss in neutralizing antibody levels for each candidate standard stored at temperatures as high as 37 °C for at least 0.46 years (candidates HPV6, 31, 33, 45, 52 and 58) or 20 °C for 3 months (candidate HPV11), indicating that the standards are stable under these real-time conditions (Supplementary Tables 14–18). The PBNA data for most of the ATD samples did not fit the Arrhenius model and no degradation rates could be calculated for the candidate standards (Table 4). The exceptions were the candidates for HPV6 and HPV52 tested by REF-2, which show a predicted loss in potency of ≤ 0.001% per year at −20 °C (Table 4).

**Table 4:** Summary of thermal degradation assessments of candidate standards HPV6, 11, 31, 33, 45, 52 and 58 tested by reference laboratories REF-1 and REF-2 using PBNA and antibody-binding methods

		Predicted loss per year at −20 °C			
Candidate Standard	Time at elevated temperatures (years)	REF-1 (PBNA)	REF-2 (PBNA)	REF-1 (Ab-Binding)	REF-2 (Ab-Binding)
HPV6	1.14	*	< 0.001%	5.23%**	0.02%
HPV11	0.25	*	*	22.3%**	Not tested
HPV31	0.46	*	*	*	0.00%
HPV33	1.64	*	*	0.14%	0.01%
HPV45	0.46	*	***	*	0.02%
HPV52	1.64	*	0.001%	0.22%	0.00%
HPV58	1.14	*	*	1.90%**	0.02%

*No or poor model fit, no prediction of loss made. **The lack of observable real-time degradation over relatively short time spans can result in predictions of unrealistically high degradation rates using Ab-binding assays. ***Estimates not obtained for HPV45 assay due to technical issues with the assay. See Supplementary Tables 14−18 for real-time stability results.

The Ab-binding data obtained by REF-2 for candidates HPV6, 31, 33, 45, 52 and 58 gave a good fit to the model for predicting degradation rates showing that the loss in potency per year for these candidates when stored at −20 °C is ≤ 0.02% (Table 4). The data obtained for the degradation samples tested in Ab-binding by REF-1 gave mixed results for predicting long-term stability. The samples stored for 1.64 years gave low predicted losses at −20 °C (candidates HPV33 and HPV52, Table 4). The data for samples stored for 0.46 years gave a poor model fit, precluding calculation of predicted loss of potency (candidates HPV31 and HPV45; Table 4). The samples stored for 1.14 years or 3 months did not give useful values which were unrealistically high (candidates HPV6 and HPV58, HPV11, Table 4). In NIBSC’s experience, particularly when the ATD data covers relatively short time periods, the model can show either no predicted degradation rate (PBNA) or an unrealistically high predicted loss (Ab-binding). Nevertheless, the real time data for these candidates show no or little difference in potencies when stored at 20 °C or 37 °C for at least 3 months (Supplementary Tables 14−18).

Overall, the data indicate that the standards are stable for long term storage at −20 °C and shipment at ambient temperatures. The stability of these standards will be monitored throughout their lifetime.

## Discussion

It is essential that HPV serology assays are validated, standardized and well-controlled to obtain reliable results. In the absence of commonly available biological reference standards, HPV serology laboratories apply their in-house reference materials to obtain user-defined “units” or titers to report results for the detection and quantification of HPV antibodies. While in-house standards can be successfully used for assuring assay performance within a laboratory, they cannot serve to harmonize results across laboratories that do not have access to the same standard.

Previous studies have shown that serology assays for HPV16 and HPV18 can be harmonized across laboratories when antibody measurements are determined relative to the WHO ISs for HPV16 and HPV18^10–12^.

Through this multi-center international collaborative study using an array of assay methods, we demonstrated that 7 candidate standards for antibodies against HPV6, 11, 31, 33, 45, 52, and 58 are suitable to serve as WHO ISs. The study included the characterization of the candidates in terms of stability, reactivity, specificity, and ability to harmonize PBNA and Ab-binding results across laboratories testing samples from both naturally infected and vaccinated individuals.

Well-controlled assays are expected to have good comparability within the laboratory. This was confirmed for most of the study participants, with satisfactory degrees of within-assay agreement observed for duplicate samples (Figs. 2 and 3) and within-laboratory agreement across independent assays (Figs. 4 and 5). However, this study also demonstrated that the assays were not standardized between laboratories, as shown by interlaboratory variability of absolute antibody measurements reported without normalization against the respective candidate standard. This high variability in estimates in absolute potencies can be attributed to the use of different methodologies and analysis procedures, different reporting units across laboratories, and the absence of a common reference standard.

To analyze the extent of inter-laboratory agreement that could be expected from the use of a common standard or reference material, the results of the HPV-antibody-positive samples were expressed relative to the relevant candidate standard (Tables 2 and 3).

The variability of results between laboratories was reduced for both PBNA and Ab-binding assays when the candidate standards were used. Many of the assay designs or analysis methods could be optimized to allow better estimation and agreement of relative potencies. Nevertheless, these values are in line with %GCVs observed in similar studies^5,11,12^. With global implementation and use of the HPV ISs in programs of continuing quality assessment and improvement, it is expected that variability between assays and laboratories will reduce over time.

Samples for testing in a WHO collaborative study may include typical samples for which the standard will be used (to assess commutability). For this study, a comprehensive assessment of commutability of the candidate standards for clinical samples tested according to specified procedures was not feasible, in part, because of the sample size and testing workload that would be required. The commutability of HPV serology reference materials might be affected by a range of factors such as sample matrix (e.g., serum, plasma, body fluid); whether antibodies have been induced by natural infection or vaccination; and whether measurement is based on neutralizing and/or binding epitopes^6^. By including plasma and serum samples from naturally infected and vaccinated individuals, some aspects of commutability was addressed here. The variability of relative potencies seen in this study are in line with observations reported in similar studies using HPV references derived from naturally infected individuals^5,11,12^.

This study demonstrated that the candidate standards for HPV6, 31, 33, 45, 52 and 58 are monospecific in reactivity against the indicated type. In addition to their use in the calibration and harmonization of assays, these standards may be used in the assessment of assay specificity. Since the HPV11 candidate standard was shown to have additional reactivity to other types, it may only be used in the calibration and harmonization of assays and not for assessing assay specificity.

The results of the collaborative study were presented to the WHO ECBS in October 2022, which agreed to the establishment of the candidates as 1st WHO IS for antibodies to HPV6 (NIBSC code 19/298), HPV11 (NIBSC code 20/174), HPV31 (NIBSC code 20/176), HPV33 (NIBSC code 19/290), HPV45 (NIBSC code 20/178), HPV52 (NIBSC code 19/296), and HPV58 (NIBSC code 19/300)^16^.

The values in IU/ampoule assigned to the ISs are given in Table 5. There is no international conventional reference measurement procedure for the quantification of HPV antibodies and the IU will not be traceable to the International System of Units (SI) of quantity. The value of the IU is arbitrary, and the uncertainty can be derived from the %CV of fill weights (see Methods). The IU between ISs of other HPV types is not comparable. However, the values of IU/ampoule assigned for these International Standards gives approximately the same order of magnitude to those assigned to the International Standards for anti-HPV16 (NIBSC code 05/134) and anti-HPV18 (NIBSC code 10/140).

**Table 5:** Establishment of 1st WHO International Standards for HPV antibodies by the Expert Committee on Biological Standardization in October 2022

1st WHO International Standard for Human Papillomavirus (HPV)	NIBSC Product code	unitage (IU/ampoule)	unitage (IU/mL) when reconstituted as directed in 0.25 mL dH_2_O	Approximate number of ampoules available from NIBSC as of Oct 2022
Type 6 antibodies	19/298	7	28	2500
Type 11 antibodies	20/174	6	24	1000
Type 31 antibodies	20/176	3	12	600
Type 33 antibodies	19/290	8	32	3000
Type 45 antibodies	20/178	2	8	700
Type 52 antibodies	19/296	14	56	900
Type 58 antibodies	19/300	20	80	2400

NIBSC HPV standards are available from the MHRA (https://nibsc.org/products/brm_product_catalogue/sub_category_listing.aspx?category=Vaccines&subcategory=Human%20Papillomavirus).

HPV laboratories that report their HPV16 and 18 serology studies in globally recognized IU/mL, rather than lab-defined titres or “units”/mL, enable their findings to be compared with other studies where results are also reported in IU^17–20^. Global utilization of the 9 ISs will enable standardization of HPV serology assays used in HPV antibody levels of unvaccinated or vaccinated individuals as part of population studies; L1-based HPV vaccine research, development, manufacturing and control; clinical trials or monitoring post licensure. Their utility is envisioned not only in light of the global strategy for cervical cancer elimination, but also in serological investigations of other HPV-related diseases and natural infections in both women and men.

The ISs, along with information of stability and instructions for storage, reconstitution, and use (which may differ between ISs) are available from the NIBSC on-line catalogue (https://nibsc.org/products/brm_product_catalogue/sub_category_listing.aspx?category=Vaccines&subcategory=Human%20Papillomavirus). The intended uses of the ISs are for the initial validation of new assays and calibration of secondary standards^8^. The routine use of calibrated secondary standards, such as those available through the Frederick National Laboratory for Cancer Research’s HPV Serology Laboratory (https://frederick.cancer.gov/research/science-areas/vaccine-immunity-and-cancer-directorate/hpv-and-covid-19-serology-laboratories), traceable to the ISs will aid in the development, validation, and standardization of HPV serology assays, as well as prevent IS depletion of limiting stocks.

## Methods

### Source materials

The process for testing, selection, and formulation of the source materials to produce the 7 candidate standards is shown in Fig. 1. Anonymized serum and plasma samples were provided to NIBSC for development into candidate materials. Donations obtained by informed consent from women naturally infected with HPV were provided by Professor Joakim Dillner, Karolinska Institute, Stockholm, Sweden, in collaboration with Dr. Jarunya Ngamkham, National Cancer Institute, Thailand (approved by the ethical and research committees of the National Cancer Institute (NCI), Thailand (EC 122/2009, decision taken 18.12.2009)^21^; Professor Mario Poljak, University of Ljubljana, Ljubljana, Slovenia (approved by the Medical Ethics Committee of the Republic of Slovenia (Consent number: 83/11/09) and also approved by the Ethical Committe of Umeå, Sweden (Nr. 118/92, 95-2400 and 98/12)^22^; and Dr. Weijin Huang, National Institutes for Food and Drug Control Beijing, P.R. China by which samples were selected from a large number of anonymized samples from a plasma center (Shanghai RAAS Blood Products Co. Ltd, Shanghai, China)^23^. Prior to development, the plasma donations were converted to serum by thrombin-treatment to cause clotting followed by defibrination by centrifugation and then filtering the supernatant. Twenty donations were initially tested by 4 external HPV reference laboratories (Supplementary Table 13) in validated PBNA and Ab-binding assays to identify the materials most suitable for development into candidate standards. Preselection criteria for development included: i) confirmation of seropositivity (as defined by each reference laboratory for their assay) to the HPV type of interest and no reactivity to other HPV vaccine types i.e., each candidate IS should be monospecific to allow assignment of clearly defined IU that is not affected by the presence of possibly cross-reactive antibodies against other HPV types.; ii) each candidate should consist of donations from at least 2 donors in order to reduce the risk that a donation with uncommon characteristics would be selected as an IS; iii) donations should be obtained from only women as data suggest that women have higher antibody responses to natural infection than men^24^.

The initial testing round identified 13 donations for development into candidate standards; however, exceptions to the selection criteria were made for the candidate antibody standards for HPV31, HPV45, and HPV11 due to the difficulty in sourcing monospecific source materials. In the case of the HPV31 and HPV45 candidate standards, only single donations were identified for development. For the candidate standard for HPV11 antibodies, only 2 donations were available for development. These were shown also to be reactive to HPV6. One of the donations was also reactive to HPV33, 52 and 58 across 1 or more methods. The cross reactivity of the HPV11 materials can be attributed, in part, to cross-reactive epitopes with other HPV types^14,25^ and/or co-infection. The use of the 2 available donations was taken as a best-case scenario for the formulation of the candidate HPV11 antibody standard.

Initial testing found low-level reactivity to at least 1 non-target HPV type in at least 1 donation for each candidate. A small-scale mixing study was performed to identify the pooling ratio for each candidate standard to mitigate the low-level, non-target reactivity while maintaining optimal seroreactivity for the target HPV type (Fig. 1). In the case of the HPV31 and HPV45 candidates, pooling was performed using HPV antibody-negative serum (Table 1, Sample E). The optimum pooling ratios for formulations were determined in a small-volume mixing study for candidate antibody standards HPV6, 31, 33, 45, 52, and 58 tested by 2 HPV reference laboratories (REF-1, REF-2 in Supplementary Tables 19−23). The HPV11 candidate standard was formulated by mixing the 2 donations without optimization.

### Production and pre-study testing of candidate International Standards

The NIBSC Human Materials Advisory Committee approved the use of the source materials for development into candidate standards (approval reference 18/07/DW). All donations used to make the candidates were tested at NIBSC and found negative for Hepatitis B surface antigen (HBsAg), antibodies to Human Immunodeficiency Virus (HIV) 1/2, and Hepatitis C (HCV) RNA. From March 2020 to June 2021, NIBSC undertook separate productions for each candidate standard. Materials were filtered, pooled and dispensed at high precision in 250 µL aliquots into glass ampoules and freeze-dried according to standard operating procedures and WHO guidelines^6^. The ampoules were sealed under 1 atmosphere of nitrogen and stored at −20 °C. Ampoule seal integrity was assessed by measuring residual oxygen by frequency modulation spectroscopy (FMS-760 from Lighthouse Instruments, Charlottesville, VA, USA) using a laser infra-red source beamed through the headspace of the ampoule. Residual moisture content was measured using the colorimetric Karl Fischer method (Mitsubishi CA-100 or CA-200, and kit obtained through A1- Envirosciences, Blyth, UK). Each candidate was assigned a unique NIBSC product code. Descriptions of the candidate ISs, including the anonymous donor identifiers, pooling information, manufacture, and validation outcomes, are provided in Supplementary Table 24. An anomaly occurred during the production of the HPV11 candidate where the material was dehydrated rather than lyophilized due to a freeze-drier failure although validation testing showed that the reactivities of all the candidate standards were similar to the results obtained during the selection process. In summary, the pre-study results confirmed the suitability of the 7 candidates for formal evaluation in the international collaborative study (Supplementary Tables 19−23).

### Sample panel

The samples distributed for testing in the collaborative study are listed in Table 1. To demonstrate that the candidate standards would be suitable for use in assays developed to monitor antibody responses in sera from individuals naturally infected with HPV as well as those vaccinated with different HPV vaccines (i.e. aspects of commutability), coded samples of each type of sample were distributed with the candidates. The HPV antibody-negative serum pool used in the mixing study was included as a negative control. HPV antibody-negative serum and sera from recipients of the 9v vaccine, were collected by Occupational Health Services at the National Institutes of Health (NIH) National Cancer Institute (NCI) at Fort Detrick, MD, under the Research Donor Protocol (RDP). Participants were healthy NCI-Frederick employees and other NIH staff that donated blood samples for in vitro research at the NCI-Frederick laboratories. The protocol is listed under NIH protocol number OH99CN046 and NCT number NCT00339911. These OHS samples were used for reagent optimization.

Pooled plasma from recipients of the 2v vaccine were provided by Dr. Simon Beddows, UK Health Security Agency, Virus Reference Department, London, UK Plasma packs were obtained from NHS Blood and Transplant, UK, Formal approval was sought from the NHS Blood and Transplant according to their own release procedures for samples considered for non-clinical use. No individual identifying information was available and no additional individual consent was required^14^.

Study samples were delivered on dry ice to participating laboratories, who were instructed to store these materials at or below -20°C.

### Study protocol

Participants were requested to use their established method(s) for the detection and quantification of antibodies to 1 or more of the 9 HPV types (HPV6, 11, 16, 18, 31, 33, 45, 52, 58). For each HPV type tested, participants were requested to test all study samples concurrently in 3 independent assays using a new set of prepared samples for each run. Participants were provided with an extra set of samples to allow for a preliminary assay run to determine optimal dilution ranges for testing. Participants were requested to set up and test at least 2 independent replicate series of dilutions (NOT 2 aliquots from a single dilution series), where practical. Written instructions and a web conference were provided to guide participants on the preparation and testing of the study samples including a recommended plate setup to ensure that certain unknown samples were assayed together to allow direct comparability.

Participants were requested to record the readout (e.g., +/−, optical density [OD], relative light units [RLU]) for each dilution and include the cut-off value indicating sero-reactivity for each assay, stating how the cut-off criteria was established and whether each sample dilution tested was considered positive or negative according to assay criteria. Participants were also requested to provide information on the preparation and use of VLPs/capsids/pseudovirions in their assay(s).

### Participants

NIBSC invited 29 HPV laboratories worldwide to participate in the collaborative study. A draft protocol was provided to the prospective participants for their review and comment. Twelve laboratories accepted the invitation with 1 subsequently withdrawing from the study. Laboratories that participated in the study are referred to by code numbers (Supplementary Table 1) allocated at random and not representing the order of listing at the end of the paper.

### Collaborative study assay methods

The assay methods performed by participants fall into 2 general categories: PBNA and Ab-binding assays. None of the participants shared a common standard operating procedure or protocol for performing the assay. The specific details of the individual assay methods used by participating laboratories are not described here to ensure the coding of the participating laboratory results remain anonymized. In general, participants performing PBNA used PsV that were prepared in-house using 293FT, 293TT or HeLaT cells. Reporter genes for measuring neutralization included those encoding for green fluorescent protein (GFP), red fluorescent protein (RFP), cyan fluorescent protein (CFP), secreted alkaline phosphatase (SEAP) and (nano)luciferase. Reporter gene readouts included fluorescence, fluorospots, OD, chemiluminescence, and RLU. PBNA cut-off values for seropositivity were defined based on the lowest dilution tested or in reference to a non-relevant PsV. Initial dilutions ranged from 1/10 to 1/100 with serial dilution steps ranging from 2-fold to 5-fold. Results were reported in neutralizing titers. Two laboratories also reported results in IU/mL traceable to the established ISs for HPV16 and HPV18 antibodies.

Participants performing Ab-binding assays described the antigen used as type-specific VLP, L1L2 VLP, pseudovirion or “antigen” with no further detail. Methods of antigen production included mammalian cell, *Pichia pastoris* and *Escherichia coli* expression systems. Readouts for Ab-binding assays included OD, mean fluorescence intensity (MFI) and RLU. Initial dilutions ranged from 1/50 to 1/100 with serial dilution steps ranging from 2-fold to 10-fold. Four laboratories used the readouts to calculate antibody-binding levels in laboratory-defined units relative to an in-house standard. In the case of HPV16 and HPV18, the same 4 laboratories reported in IU/mL. Four laboratories reported their cut-off values for sample seropositivity with 2 laboratories reporting that the cut-off values were determined using sera from children. Two laboratories reported endpoint titers with no cut-off defined for seropositivity.

### Statistical methods

Statistical analysis of assay results within and between laboratories is based on the general principles for establishing International Standards and other biological reference materials for serology^6,26–28^.

Samples were scored as positive ‘P’ or negative ‘N’ for antibodies based on the criteria defined by the participating laboratories. For PBNA, if no cut-off for seropositivity was provided, a sample was scored negative if the titer was less than the lowest dilution tested. For Ab-binding assays, samples were not scored if the cut-off was not defined. An overall laboratory score was assigned to each sample according to the majority response reported across independent assays (e.g., 2 out of 3 assays). Data was assessed further if the sample scored seropositive by > 50% of laboratories. At this point, the Ab-binding levels reported by the 2 laboratories with no defined cutoffs were included in the analysis.

Overall antibody concentrations, i.e., titers or “units”/mL, for each sample were calculated as the median across the independent assays for each laboratory, method, and HPV type.

Intra-assay variability was assessed by calculating the ratios of median antibody concentration for duplicate samples I & P, A & J, B & K (Table 1). A ratio of 1 indicated matching results. Ratios less than 0.8 or greater than 1.20 (i.e., more than 20% difference in measurements) were taken as higher variability. A ratio of 0 indicated that 1 of the duplicate samples was scored negative by the laboratory. Intra-assay variability of results for antibodies against HPV6 and 11 (and 58 in PBNA only) was not assessed due to the limited availability of seropositive samples for inclusion in the study.

Inter-assay variability within each laboratory was assessed by calculating the ratio of the maximum and minimum antibody concentration (un-transformed) reported for each sample across independent assays. A > 4-fold difference in the maximum and minimum concentrations (Max:Min) was selected as an indicator of greater intra-laboratory variability.

Overall mean results across laboratories were calculated for each sample as the GM of the laboratory median antibody concentrations or potencies expressed relative to the indicated candidate standard. For the purpose of this assessment, candidate standards were assigned a value of 1 to determine overall GM relative potencies. The between laboratory (inter-laboratory) variability was assessed by calculating GCV using the equation GCV= [10^s^-1] × 100% where s is the standard deviation of the log_10_ transformed median values. Ratios of the maximum and minimum results (un-transformed) across laboratories were also calculated.

### Thermal stability assessment

An accelerated thermal degradation (ATD) study was performed in order to predict the long-term stability of the candidate standards when stored at the recommended temperature of −20 °C. Ampoules of the candidate standards were stored at −70 °C, −20 °C, 4 °C, 20 °C, 37 °C and 45 °C and then removed at indicated time points and held at −70 °C or below until assayed. The ATD samples were shipped on dry ice to 2 HPV reference laboratories (REF-1 and REF-2) for testing in PBNA and Ab-binding assays. The ATD samples were tested concurrently against the respective baseline sample (−70 °C or −20 °C) in at least 3 independent assays. Estimates of antibody concentration of the ATD samples (titers for PBNA, units/ml for Ab-binding) were reported to NIBSC for analysis. GM potencies and 95% confidence limits for the ATD samples stored at the elevated temperatures were calculated relative to the material stored at the baseline temperature (−70 °C or −20 °C). The baseline samples were assigned a value of 1 for the purpose of the assessment. To predict the degradation rate of the candidates when stored at −20 °C, the relative potencies of the ATD samples were used to fit an Arrhenius equation assuming first-order decay^15^.

## Supplementary information

Supplementary Information

## Data Availability

Median laboratory-reported antibody concentrations obtained in PBNA and ELISA are listed in Supplementary Tables 3-11. Raw data can be made available upon request, but the participant’s name will be anonymized.
