# Supplementary material for: WHO International Standards for antibodies to HPV6 HPV11 HPV31 HPV33 HPV45 HPV52 and HPV58
Source: NPJ Vaccines. 2024 Sep 10;9:165. doi: 10.1038/s41541-024-00949-2 (PMC11387505; doi:10.1038/s41541-024-00949-2)
Supplement: Supplementary file 1 — Supplementary Information [file 41541_2024_949_MOESM1_ESM.pdf]

WHO International Standards for antibodies to HPV6 HPV11 HPV31 HPV33 HPV45 HPV52 and HPV58

SUPPLEMENTARY TABLES

Supplementary Table 1. Distribution of antibody testing of samples across participating laboratories*

Laboratory Code	1	2	3	4	5	6	7	8	9	10	11
PBNA			+	+	+	+	+	+	+	+	+
Ab-Binding	+	+				+	+	+		+	

*Laboratories tested for antibodies to the 9 vaccine types except for Lab-2, which tested for HPV6, 11, 16 and 18, and Lab-9, which tested for HPV18 and HPV45. + indicates the method used for testing.

.

Supplementary Table 2. Assessment of intra-assay variability. Ratios of results in terms of absolute median antibody concentration for duplicate samples (I:P, A:J and B:K) in PBNA and Ab-binding assays for HPV6, 11, 16, 18, 31, 33, 45, 52 and 58 antibodies. A ratio of 1.00 indicates that the duplicates had matching results. A greater than 20% difference in results for duplicate samples was selected as a guideline to identify greater variability.

Assay method	Lab Code	HPV 6 I:P	HPV 11 I:P	HPV 16 I:P	HPV 18 I:P	HPV 31 I:P	HPV 33 I:P	HPV 45 I:P	HPV 52 I:P	HPV 58 I:P	HPV 33 A:J	HPV 52 B:K
PBNA	9	na	na	-	***0.45***	-	-	<	-	na	-	-
PBNA	7	na	na	1.01	0.94	***0.64***	1.00	1.20	<	na	1.11	**1.29**
PBNA	10	na	na	1.11	0.94	***0.64***	1.18	***1.47***	<	na	0.93	1.05
PBNA	5	na	na	1.00	1.00	1.00	1.00	***3.00***	<	na	1.00	1.00
PBNA	4	na	na	0.84	0.96	0.92	0.83	**0.70**	0.89	na	1.02	1.18
PBNA	8	na	na	1.20	1.06	***0.61***	<	<	<	na	0.92	0.90
PBNA	6	na	na	***0.50***	1.00	1.00	1.00	1.00	1.00	na	1.00	1.00
PBNA	3	na	na	***1.31***	1.03	0.94	0.91	1.05	1.02	na	1.09	0.85
PBNA	11	na	na	0.97	1.19	1.06	1.10	1.12	1.11	na	***0.76***	0.97
Ab-Binding	7	na	na	0.99	1.03	1.04	1.08	1.01	1.02	1.07	0.93	1.05
Ab-Binding	2	na	na	***0.71***	1.16	-	-	-	-	-	-	-
Ab-Binding	1	na	na	0.97	0.99	0.84	**0.70**	1.09	<	0.83	0.92	1.20
Ab-Binding	10	na	na	0.97	0.97	1.04	1.03	0.89	0.92	0.98	0.94	1.13
Ab-Binding	8	na	na	***1.39***	1.07	1.17	0.97	0.96	***0.77***	0.86	1.07	***1.44***
Ab-Binding	6	na	na	***0.75***	***3.75***	***0.75***	***0.50***	1.00	1.00	1.00	***0.50***	***0.50***

<, At least one analyte (I or P) was reported below the assay cut-off. **-**, assay not performed. ***Bold*** indicates a >20% difference in duplicate results. **na**, not assessed as ≥ 50% of laboratories scored the samples negative.

Supplementary Table 3. HPV6 assays: Study samples scored as positive or negative for HPV antibodies in Ab-binding assays and PBNA. Laboratory median estimated antibody concentrations and relative potencies are listed for samples scored overall positive.

Sample		X	*X*	A	B	C	D	E	**F**	G	*G*	H	*H*	I	J	K	L	M	N	O	P
**HPV6 Assay**	Lab Code	9V vaccinee Reference	*Relative to F*	HPV33 IS 19/290	HPV52 IS 19/296	Natural HPV33	Natural HPV52	Neg	**HPV6 IS 19/298**	HPV11 IS 20/174	*Relative to F*	Natural HPV6	*Relative to F*	2v vaccinee Reference	HPV33 IS 19/290	HPV52 IS 19/296	HPV58 IS 19/300	Natural HPV68	HPV31 IS 20/176	HPV45 IS 20/178	2v vaccinee Reference
Ab-Binding (units/mL or titer)	7	P 968	*27.69*	N	N	N	N	N	**P 35**	N	na	P 33	*0.93*	P 23	N	N	N	N	N	N	P 22
	2	* 9941	*3.97*	*	*	*	*	*	*** 2504**	* 3823	na	* 2162	*0.86*	*	*	*	*	*	*	*	*
	1	P 122	*64.21*	N	N	N	N	N	**P 1.9**	P/N	na	P 1.4	*0.74*	P/N	N	N	N	N	N	N	P 0.3
	10	P 121	*49.39*	N	N	N	N	N	**P 2.5**	N	na	P 2.0	*0.83*	P/N 0.8	N	N	N	N	N	N	P/N
	8	P 120	*nc*	N	N	N	N	N	**N**	N	na	N	*nc*	N	N	N	N	N	N	N	N
	6	* 32000	*10.00*	*	*	*	*	*	*** 3200**	*	na	* 3200	*1.00*	*	*	*	*	*	*	*	*
PBNA (titer)	7	P 4607	*32.11*	N	N	P/N	N	N	**P 144**	P/N 38	*0.26*	P 72	*0.50*	N	N	N	N	N	N	N	N
	10	P 37258	*36.12*	N	N	N	N	N	**P 1032**	P 339	*0.33*	P 483	*0.47*	N	N	N	N	P/N	N	N	N
	5	P 8100	*18.00*	N	N	N	N	N	**P 450**	P 50	*0.11*	P 50	*0.11*	N	N	N	N	N	N	N	N
	4	P 6604	*23.18*	N	N	N	N	N	**P 285**	N	*nc*	P 150	*0.53*	N	N	N	N	N	N	N	N
	8	P 15215	*94.13*	N	N	N	N	N	**P 162**	N	*nc*	P 98	*0.60*	N	N	N	N	N	N	N	N
	6	P 1280	*16.00*	N	N	N	N	N	**P 80**	N	*nc*	P 40	*0.50*	N	N	N	N	N	N	N	N
	3	P 7651	*31.10*	N	P/N	P 37	N	P/N 28	**P 246**	P 70	*0.29*	P 147	*0.60*	N	N	P/N	N	P 28	P/N 23	P/N	N
	11	P 45979	*86.59*	N	P 58	P 73	N	P 52	**P 531**	P 119	*0.22*	P 171	*0.32*	N	N	P 59	P/N	P 69	P 61	P 64	N

Abbreviations/Notes: **P**, positive (no shading). **N**, negative (dark grey). **P/N**, both positive and negative results were obtained (light grey: underline indicates overall result). *****, Lab-2 and Lab-6 cut-off not established**-** sample not scored. Titers are shown for Lab-2 and Lab-6 where samples are consensus-positive across laboratories. Column in **bold type** highlights the results for the candidate International Standard for HPV6 Ab. Values in *Italics* indicate potencies relative to the candidate International Standard for HPV6 Ab (Sample F). **na**, not applicable; **nc**, not calculated-sample and/or candidate IS scored negative.

Supplementary Table 4. HPV11 assays: Study samples scored as positive or negative for HPV antibodies in Ab-binding assays and PBNA. Laboratory median estimated antibody concentrations and relative potencies are listed for samples scored overall positive.

Sample		X	*X*	A	B	C	D	E	F	**G**	H	I	J	K	L	M	N	O	P
**HPV11 Assay**	Lab Code	9V vaccinee Reference	*Relative to G*	HPV33 IS 19/290	HPV52 IS 19/296	Natural HPV33	Natural HPV52	Neg	HPV6 IS 19/298	**HPV11 IS 20/174**	Natural HPV6	2v vaccinee Reference	HPV33 IS 19/290	HPV52 IS 19/296	HPV58 IS 19/300	Natural HPV68	HPV31 IS 20/176	HPV45 IS 20/178	2v vaccine e Referen ce
Ab-Binding (units/mL or titer)	7	P<br>1165	*71.52*	N	P/N	P/N	N	N	P/N<br>2	**P<br>16**	P<br>3	P/N	N	N	N	N	N	N	N
	2	*<br>51170	*32.53*	*	*	*	*	*	*	***<br>1573**	*	*	*	*	*	*	*	*	*
	1	P<br>116	*165.71*	N	N	N	N	N	N	**P<br>0.7**	N	N	N	N	N	N	N	N	N
	10	P<br>237	*65.29*	N	N	N	N	N	N	**P<br>3.6**	N	P/N<br>1	N	N	N	N	N	N	P/N<br>1
	8	P<br>353	*nc*	N	N	N	N	N	N	**N**	N	N	N	N	N	N	N	N	N
	6	*<br>64000	*40.00*	*	*	*	*	*	*	***<br>1600**	*	*	*	*	*	*	*	*	*
PBNA (titer)	7	P<br>3334	*47.28*	N	N	N	N	N	N	**P<br>71**	N	N	N	N	N	N	N	N	N
	10	P<br>100084	*73.46*	P/N	N	P/N<br>110	N	N	N	**P<br>1363**	N	N	P/N	N	N	N	N	N	N
	5	P<br>24300	*162.00*	N	N	N	N	N	N	**P<br>150**	N	N	N	N	N	N	N	N	N
	4	P<br>3704	*nc*	N	N	N	N	N	N	**N**	N	N	N	N	N	N	N	N	N
	8	P<br>34546	*71.61*	N	N	P<br>100	N	P/N	N	**P<br>482**	N	N	N	N	N	P<br>91	N	N	N
	6	P<br>1280	*nc*	N	N	N	N	N	N	**N**	N	N	N	N	N	N	N	N	N
	3	P<br>6951	*71.83*	N	N	P<br>21	N	N	N	**P<br>97**	N	N	N	N	N	N	N	N	N
	11	P<br>37350	*115.63*	P/N	N	P<br>42	P/N<br>43	N	N	**P<br>323**	N	P/N	P/N<br>41	N	P<br>52	N	N	N	P/N<br>55

Abbreviations/Notes: **P**, positive (no shading). **N**, negative (dark grey). **P/N**, both positive and negative results were obtained (light grey: underline indicates overall result). *****, Lab-2 and Lab-6 cut-off not established- sample not scored. Titers are shown for Lab-2 and Lab-6 where samples are consensus-positive across laboratories. Column in **bold type** highlights the results for the candidate International Standard for HPV11 Ab. Values in *Italics* indicate potencies relative to the candidate International Standard for HPV11 Ab (Sample G). **nc**, not calculated. Sample and/or candidate IS scored negative.

Supplementary Table 5. HPV16 assays: Study samples scored as positive or negative for HPV antibodies in Ab- binding assays and PBNA. Laboratory median estimated antibody concentrations are listed for samples scored overall positive.

Sample		X	A	B	C	D	E	F	G	H	I	J	K	L	M	N	O	P
**HPV16 Assay**	Lab Code	9V vaccinee Reference	HPV33 IS 19/290	HPV52 IS 19/296	Natural HPV33	Natural HPV52	Neg	HPV6 IS 19/298	HPV11 IS 20/174	Natural HPV6	2v vaccinee Reference	HPV33 IS 19/290	HPV52 IS 19/296	HPV58 IS 19/300	Natural HPV68	HPV31 IS 20/176	HPV45 IS 20/178	2v vaccinee Reference
Ab-Binding (units/mL or titer)	7	P 489	N	N	N	N	N	N	N	N	P 3315	N	N	N	N	N	N	P 3347
	2	* 38691	*	*	*	*	*	*	*	*	* 93103	*	*	*	*	*	*	* 131169
	1	P 846.5	N	N	N	N	N	N	N	N	P 7956	N	N	N	N	N	N	P 8225
	10	P 413	N	N	N	N	N	N	N	N	P 2313	N	N	N	N	N	N	P 2385
	8	P 486	N	N	N	N	N	N	N	N	P 3375	N	N	N	N	N	N	P 2422
	6	* 64000	*	*	*	*	*	*	*	*	* 192000	*	*	*	*	*	*	* 256000
PBNA (titer)	7	P 1211	N	N	N	N	N	N	N	N	P 3771	N	N	N	N	N	N	P 3733
	10	P 40836	N	N	N	N	N	N	N	N	P 134130	N	N	N	<u>P</u>/N 120	N	N	P 120774
	5	P 24300	N	N	N	N	N	N	N	P/<u>N</u>	P 45000	N	N	N	P/<u>N</u>	N	N	P 45000
	4	P 10111	N	N	N	N	N	N	N	N	P 34046	N	N	N	N	N	N	P 40630
	8	P 16397	N	N	N	N	N	N	N	N	P 59540	N	N	N	N	N	N	P 49795
	6	P 20480	N	N	N	N	N	N	N	N	P 81920	N	N	N	P 40	N	N	P 163840
	3	P 15818	N	N	N	N	N	N	N	N	P 51545	N	N	N	P 30	N	N	P 39322
	11	P 109482	N	N	N	N	N	N	N	<u>P</u>/N 43	P 290678	N	N	N	<u>P</u>/N 63	N	N	P 299516

Abbreviations/Notes: **P**, positive (no shading). **N**, negative (dark grey). **P/N**, both positive and negative results were obtained (light grey. <u>underline</u> indicates overall result). *, Lab-2 and Lab-6 cut-off not established- sample not scored. Titers are shown for Lab-2 and Lab-6 where samples are consensus-positive across laboratories.

Supplementary Table 6. HPV18 assays: Study samples scored as positive or negative for HPV antibodies in Ab-binding assays and PBNA. Laboratory median estimated antibody concentrations are listed for samples scored overall positive.

Sample		X	A	B	C	D	E	F	G	H	I	J	K	L	M	N	O	P
**HPV18 Assay**	Lab Code	9V vaccinee Reference	HPV33 IS 19/290	HPV52 IS 19/296	Natural HPV33	Natural HPV52	Neg	HPV6 IS 19/298	HPV11 IS 20/174	Natural HPV6	2v vaccinee Reference	HPV33 IS 19/290	HPV52 IS 19/296	HPV58 IS 19/300	Natural HPV68	HPV31 IS 20/176	HPV45 IS 20/178	2v vaccinee Reference
Ab-Binding (units/mL or titer)	7	P<br>183	N	N	P/N	N	N	N	N	N	P<br>794	N	N	N	N	N	N	P<br>770
	2	*<br>10187	*	*	*	*	*	*	*	*	*<br>20502	*	*	*	*	*	*	*<br>17666
	1	P<br>52	N	N	N	N	N	N	N	N	P<br>131	N	N	N	N	N	N	P<br>132
	10	P<br>232	N	N	N	N	N	N	N	N	P<br>844	N	N	N	N	N	N	P<br>869
	8	P<br>376	N	N	N	N	N	N	N	N	P<br>1524	N	N	N	N	N	N	P<br>1423
	6	*<br>32000	*	*	*	*	*	*	*	*	*<br>48000	*	*	*	*	*	*	*<br>12800
PBNA (titer)	9	P<br>23156	N	N	N	N	N	N	N	N	P<br>83547	N	N	N	N	N	N	P<br>185000
	7	P<br>394	N	N	N	N	N	N	N	N	P<br>1647	N	N	N	N	N	N	P<br>1754
	10	P<br>12150	N	N	N	N	N	N	N	N	P<br>44729	N	N	N	N	N	N	P<br>47710
	5	P<br>2700	N	N	N	N	N	N	N	N	P<br>15000	N	N	N	N	N	N	P<br>15000
	4	P<br>2979	N	N	N	N	N	N	N	N	P<br>11617	N	N	N	N	N	N	P<br>12046
	8	P<br>4903	N	N	N	N	N	N	N	N	P<br>18943	N	N	N	N	N	N	P<br>20775
	6	P<br>10240	N	N	N	N	N	N	N	N	P<br>81920	N	N	N	N	N	N	P<br>81920
	3	P<br>2390	N	N	N	N	N	N	N	N	P<br>10549	N	N	N	N	N	N	P<br>10244
	11	P<br>10592	N	N	N	N	N	N	N	N	P<br>35570	N	N	N	N	N	N	P<br>29767

Abbreviations/Notes: **P**, positive (no shading). **N**, negative (dark grey). **P/N**, both positive and negative results were obtained (light grey. underline indicates overall result). *****, Lab-2 and Lab-6 cut-off not established**-** sample not scored. Titers are shown for Lab-2 and Lab-6 where samples are consensus-positive across laboratories.

Supplementary Table 7. HPV31 assays: Study samples scored as positive or negative for HPV antibodies in Ab-binding assays and PBNA. Laboratory median antibody concentrations are listed for samples scored overall positive.

Sample		X	*X*	A	B	C	*C*	D	E	F	G	H	I	*I*	J	K	L	M	**N**	O	P	*P*
**HPV31 Assay**	Lab Code	9V vaccinee Reference	*Relative to N*	HPV33 IS 19/290	HPV52 IS 19/296	Natural HPV33	*Relative to N*	Natural HPV52	Neg	HPV6 IS 19/298	HPV11 IS 20/174	Natural HPV6	2v vaccinee Reference	*Relative to N*	HPV33 IS 19/290	HPV52 IS 19/296	HPV58 IS 19/300	Natural HPV68	**HPV31 IS 20/176**	HPV45 IS 20/178	2v vaccinee Reference	*Relative to N*
Ab-Binding (units/mL or titer)	7	P		N	N	N		N	N	N	P/N	N	P		N	N	N	N	**P**	N	P	
		1118	*42.87*				na				5		298	*11.42*					**26**		287	*11.01*
	1	P		N	N	N		N	N	N	N	N	P		N	N	N	N	**P**	N	P	
		120	*100.00*				na						16	*13.33*					**1.2**		19	*15.83*
	10	P		N	N	N		N	N	N	N	N	P		N	N	N	N	**P**	N	P	
		428	*44.93*				na						73	*7.66*					**10**		70	*7.40*
	8	P		N	N	N		N	N	N	N	N	P		N	N	N	N	**P/N**	N	P	
		321	*nc*				na						71	*nc*							61	*nc*
	6	*		*	*	*		*	*	*	*	*	*		*	*	*	*	*	*	*	
		32000	*40.00*				na						12000	*15.00*					800		16000	*20.00*
PBNA (titer)	7	P		N	N	P/N		N	N	N	N	N	P		N	N	N	N	**P**	N	P	
		5098	*52.02*			50	*0.51*						273	*2.79*					**98**		427	*4.36*
	10	P		N	N	P		N	N	N	N	N	P		N	N	N	N	**P**	N	P	
		18686	*100.73*			161	*0.87*						809	*4.36*					**186**		1268	*6.84*
	5	P		N	N	P		N	N	N	N	N	P		N	N	N	N	**P**	N	P	
		8100	*162.00*			50	*1.00*						450	*9.00*					**50**		450	*9.00*
	4	P		N	N	N		P/N	N	N	P/N	N	P		N	N	N	N	**P**	N	P	
		4093	*21.67*				*nc*						1044	*5.53*					**189**		1138	*6.02*
	8	P		N	N	P		N	N	N	N	N	P		N	N	N	N	**P/N**	N	P	
		6668	*nc*			89	*nc*						215	*nc*							355	*nc*
	6	P		N	N	N		N	N	N	P/N	N	P		N	N	N	N	**P**	N	P	
		2560	*16.00*				*nc*						1280	*8.00*					**160**		1280	*8.00*
	3	P		N	N	P		N	N	N	N	N	P		N	N	N	N	**P**	N	P	
		5755	*71.41*			58	*0.72*						301	*3.74*					**81**		320	*3.98*
	11	P		N	N	P		N	N	N	N	N	P		N	N	N	N	**P**	N	P	
		14907	*138.03*			79	*0.73*						608	*5.63*					**108**		573	*5.31*

Abbreviations/Notes: **P**, positive (no shading). **N**, negative (dark grey). **P/N**, both positive and negative results were obtained (light grey: underline indicates overall result). *****, Lab-6 cut-off not established- sample not scored. Titers are shown for Lab-6 where samples are consensus-positive across laboratories. Column in **bold type** highlights the results for the candidate International Standard for HPV31 Ab. Values in *Italics* indicate potencies relative to the candidate International Standard for HPV31 Ab (Sample N). na, not applicable; nc, not calculated-sample and/or candidate IS scored negative.

Supplementary Table 8. HPV33 assays: Study samples scored as positive or negative for HPV antibodies in Ab-binding assays and PBNA. Laboratory median estimated antibody concentrations are listed for samples scored overall positive. **Supplementary Table 8 continued on next page.**

Sample		X	*X*	**A**	B	C	*C*	D	E	F	G	*G*	H
**HPV33 Assay**	Lab Code	9V vaccinee Reference	*Relative to A*	**HPV33 IS 19/290**	HPV52 IS 19/296	Natural HPV33	*Relative to A*	Natural HPV52	Neg	HPV6 IS 19/298	HPV11 IS 20/174	*Relative to A*	Natural HPV6
Ab-Binding (units/mL or titer)	7	P		**P**	P/N	P		P/N	N	N	P		N
		643	*28.87*	**22**		51	*2.29*	1.7			2.1	na	
	1	P		**P**	N	P		N	N	N	N		N
		119	*91.54*	**1.3**		3.9	*3.00*					na	
	10	P		**P**	N	P		N	N	N	N		N
		1112	*41.80*	**27**		94	*3.52*					na	
	8	P		**P**	N	P		N	N	N	N		N
		704	*95.29*	**7.4**		41	*5.53*					na	
	6	*		*	*	*		*	*	*	*		*
		64000	*80.00*	**800**		2000	*2.50*					na	200
PBNA (titer)	7	P		**P**	N	P		N	N	N	P		N
		6808	*56.73*	**120**		265	*2.21*				90	0.75	
	10	P		**P**	N	P		N	N	N	P		N
		36506	*67.57*	**540**		1056	*1.96*				348	0.64	
	5	P		**P**	N	P		N	N	N	P		N
		24300	*54.00*	**450**		450	*1.00*				150	0.33	
	4	P		**P**	N	P		P/N	N	N	P		N
		20560	*43.90*	**468**		865	*1.85*				113	0.24	
	8	P		**P**	N	P		P	N	P/N	P		P/N
		15774	*122.06*	**129**		322	*2.50*				105	0.81	
	6	P		**P**	N	P		P	N	N	P		N
		20480	*64.00*	**320**		1280	*4.00*				160	0.50	
	3	P		**P**	N	P		P	N	N	P		N
		17734	*96.64*	**184**		475	*2.59*				119	0.65	
	11	P		**P**	N	P		N	N	N	P		N
		99635	*113.87*	**875**		2003	*2.29*				366	0.42	

Abbreviations/Notes: **P**, positive (no shading). **N**, negative (dark grey). **P/N**, both positive and negative results were obtained (light grey: underline indicates overall result). *****, Lab-6 cut-off not established- sample not scored. Titers are shown for Lab-6 where samples are consensus-positive across laboratories. Column in **bold type** highlights the results for the candidate International Standard for HPV33 Ab. Values in *Italics* indicate potencies relative to the candidate International Standard for HPV33 Ab (Sample A). **na**, not applicable; **nc**, not calculated. Sample and/or candidate IS scored negative. **Supplementary Table 8. continued**

Supplementary Table 8 continued. HPV33 assays: Study samples scored as positive or negative for HPV antibodies in Ab-binding assays and PBNA. Laboratory median estimated antibody concentrations are listed for samples scored overall positive.

Sample		I	*I*	**J**	*J*	K	L	M	N	O	P	*P*
**HPV33 Assay**	Lab Code	2v vaccinee Reference	*Relative to A*	**HPV33 IS 19/290**	*Relative to A*	HPV52 IS 19/296	HPV58 IS 19/300	Natural HPV68	HPV31 IS 20/176	HPV45 IS 20/178	2v vaccinee Reference	*Relative to A*
Ab-Binding (units/mL or titer)	7	P		**P**		P/N	N	N	N	N	P	
		23	*1.05*	**21**	*0.93*						22	*0.97*
	1	P		**P**		N	N	N	N	N	P	
		0.7	*0.54*	**1.2**	*0.92*						1.0	*0.77*
	10	P		**P**		N	N	N	N	N	P	
		24	*0.91*	**25**	*0.94*						24	*0.89*
	8	P		**P/N**		N	N	N	N	N	P	
		16	*2.11*	**7.9**	*1.07*						16	*2.18*
	6	*		*		*	*	*	*	*	*	
		6400	*8.00*	**400**	*0.50*						12800	*16.00*
PBNA (titer)	7	P		**P**		N	N	N	N	N	P	
		30	*0.25*	**133**	*1.11*						30	*0.25*
	10	P		**P**		N	N	N	N	N	P/N	
		129	*0.24*	**502**	*0.93*						109	*0.20*
	5	P		**P**		N	N	N	N	N	P	
		50	*0.11*	**450**	*1.00*						50	*0.11*
	4	P		**P**		N	N	N	N	N	P	
		152	*0.32*	**479**	*1.02*						184	*0.39*
	8	P/N		**P**		N	N	N	N	N	P/N	
			na	**118**	*0.92*						51	*0.39*
	6	P		**P**		N	N	P/N	N	N	P	
		160	*0.50*	**320**	*1.00*						160	*0.50*
	3	P		**P**		N	N	N	N	N	P	
		40	*0.22*	**199**	*1.09*						44	*0.24*
	11	P		**P**		P/N	N	N	N	N	P	
		310	*0.35*	**668**	*0.76*						283	*0.32*

Supplementary Table 8. Continued. *Abbreviations/Notes*: **P**, positive (no shading). **N**, negative (dark grey). **P/N**, both positive and negative results were obtained (light grey: underline indicates overall result). *****, Lab-6 cut-off not established- sample not scored. Titers are shown for Lab-6 where samples are consensus-positive across laboratories. Column in **bold type** highlights the results for the candidate International Standard for HPV33 Ab. Values in *Italics* indicate potencies relative to the candidate International Standard for HPV33 Ab (Sample A). **na**, not applicable; **nc**, not calculated. Sample and/or candidate IS scored negative.

Supplementary Table 9. HPV45 assays: Study samples scored as positive or negative for HPV antibodies in Ab-binding assays and PBNA. Laboratory median estimated antibody concentrations are listed for samples scored overall positive.

Sample		X	*X*	A	B	C	D	E	F	G	H	I	*I*	J	K	L	M	N	**O**	P	*P*
**HPV45 Assay**	Lab Code	9V vaccinee Reference	*Relative to O*	HPV33 IS 19/290	HPV52 IS 19/296	Natural HPV33	Natural HPV52	Neg	HPV6 IS 19/298	HPV11 IS 20/174	Natural HPV6	2v vaccinee Reference	*Relative to O*	HPV33 IS 19/290	HPV52 IS 19/296	HPV58 IS 19/300	Natural HPV68	HPV31 IS 20/176	**HPV45 IS 20/178**	2v vaccinee Reference	*Relative to O*
Ab-Binding (units/mL or titer)	7	P		N	N	N	N	N	N	N	N	P		N	N	N	N	N	**P**	P	
		643	*20.39*									231	*7.33*						**32**	229	*7.25*
	1	P		N	N	N	N	N	P	N	P	P		N	N	N	N	N	**P**	P	
		139	*73.16*						3		3	35	*18.42*						**1.9**	32	*16.84*
	10	P		N	N	N	N	N	N	N	N	P		N	N	N	N	N	**P**	P	
		304	*15.05*									56	*2.80*						**20**	63	*3.13*
	8	P		N	N	N	N	N	N	N	N	P		N	N	N	N	N	**P**	P	
		152	*20.35*									42	*5.63*						**7**	44	*5.85*
	6	*		*	*	*	*	*	*	*	*	*		*	*	*	*	*	*	*	
		16000	*20.00*									12800	*16.00*						**800**	12800	*16.00*
PBNA (titer)	9	P		N	N	P/<u>N</u>	N	P/<u>N</u>	N	P/<u>N</u>	N	<u>P</u>/N		N	N	N	N	N	**P/<u>N</u>**	P/<u>N</u>	
		18308	*nc*									30	*nc*								*nc*
	7	P		N	N	N	N	N	N	N	N	P		N	N	N	N	N	**P**	P	
		5071	*82.46*									88	*1.42*						**62**	73	*1.19*
	10	P		N	N	N	N	N	N	N	N	P		N	N	N	N	N	**P**	P	
		24828	*75.47*									326	*0.99*						**329**	222	*0.67*
	5	P		N	N	N	N	N	N	N	N	P		N	N	N	N	N	**P**	P	
		8100	*162.00*									150	*3.00*						**50**	50	*1.00*
	4	P		N	N	N	N	N	N	N	N	<u>P</u>/N		N	N	N	N	N	**N**	<u>P</u>/N	
		4166	*nc*									136	*nc*							196	*nc*
	8	P		N	N	N	N	N	N	N	N	N		N	N	N	N	N	**P/<u>N</u>**	N	
		5047	*nc*										*nc*								*nc*
	6	P		N	N	N	N	N	N	N	N	P		N	N	N	N	N	**P**	P	
		1280	*16.00*									160	*2.00*						**80**	160	*2.00*
	3	P		N	N	N	N	N	N	N	N	P		N	N	N	N	N	**P**	P	
		5043	*70.66*									48	*0.68*						**71**	46	*0.64*
	11	P		N	N	N	N	N	N	N	N	P		N	N	N	N	N	**P**	P	
		15582	*115.42*									171	*1.27*						**135**	153	*1.13*

Abbreviations/Notes: **P**, positive (no shading). **N**, negative (dark grey). **P/N**, both positive and negative results were obtained (light grey: <u>underline</u> indicates overall result). *****, Lab-6 cut-off not established- sample not scored. Titers are shown for Lab-6 where samples are consensus-positive across laboratories. Column in **bold type** highlights the results for the candidate International Standard for HPV45 Ab. Values in *Italics* indicate potencies relative to the candidate International Standard for HPV45 Ab (Sample O). **nc**, not calculated. Sample and/or candidate IS scored negative.

Supplementary Table 10. HPV52 assays: Study samples scored as positive or negative for HPV antibodies in Ab-binding assays and PBNA. Laboratory median estimated antibody concentrations are listed for samples scored overall positive. **Supplementary Table 10 continued on next page.**

Sample		X	*X*	A	**B**	C	*C*	D	*D*	E	F	G	*G*	H
**HPV52 Assay**	Lab Code	9V vaccinee Reference	*Relative to B*	HPV33 IS 19/290	**HPV52 IS 19/296**	Natural HPV33	*Relative to B*	Natural HPV52	*Relative to B*	Neg	HPV6 IS 19/298	HPV11 IS 20/174	*Relative to B*	Natural HPV6
Ab-Binding (units/mL or titer)	7	P		N	**P**	N		N		N	N	N		N
		813	*14.73*		**55**		*nc*		*na*				*na*	
	1	P		N	**P**	N		N		N	N	N		N
		120	*18.11*		**6.6**		*nc*		*na*				*na*	
	10	P		N	**P**	P		N		N	N	N		N
		539	*21.52*		**25**	3.1	*0.12*		*na*				*na*	
	8	P		N	**P**	P		N		N	N	N		N
		268	*18.60*		**14**	3.0	*0.21*		*na*				*na*	
	6	*		*	*	*		*		*	*	*		*
		32000	*16.00*		**2000**	800	*0.40*		*na*				*na*	
PBNA (titer)	7	P		N	**P**	P		P		N	N	<u>P</u>/N		N
		5498	*10.21*		**539**	97	*0.18*	61	*0.11*			21	*0.04*	
	10	P		N	**P**	P		P		N	N	P		N
		20700	*17.27*		**1199**	238	*0.20*	370	*0.31*			108	*0.09*	
	5	P		N	**P**	P		N		N	N	<u>P</u>/N		N
		8100	*18.00*		**450**	150	*0.33*		*nc*			50	*0.11*	
	4	P		N	**P**	N		P		N	N	N		N
		1774	*13.57*		**131**		*nc*	114	*0.87*				*nc*	
	8	P		N	**P**	P		P		N	N	P		N
		20140	*38.28*		**526**	189	*0.36*	66	*0.13*			55	*0.10*	
	6	P		N	**P**	P		P		P/<u>N</u>	P/<u>N</u>	P		N
		5120	*8.00*		**640**	160	*0.25*	320	*0.50*			80	*0.13*	
	3	P		N	**P**	P		P		<u>P</u>/N	N	P		N
		6399	*24.26*		**264**	76	*0.29*	66	*0.25*	22		65	*0.25*	
	11	P		N	**P**	P		P		N	N	<u>P</u>/N		N
		13103	*16.86*		**777**	143	*0.18*	202	*0.26*			57	*0.07*	

Abbreviations/Notes: **P**, positive (no shading). **N**, negative (dark grey). **P/N**, both positive and negative results were obtained (light grey: <u>underline</u> indicates overall result). *****, Lab-6 cut-off not established- sample not scored. Titers are shown for Lab-6 where samples are consensus-positive across laboratories. Column in **bold type** highlights the results for the candidate International Standard for HPV52 Ab. Values in *Italics* indicate potencies relative to the candidate International Standard for HPV52 Ab (Sample B). **na**, not applicable; **nc**, not calculated. Sample and/or candidate IS scored negative. **Supplementary Table 10 continued**.

Supplementary Table 10 continued. HPV52 assays: Study samples scored as positive or negative for HPV antibodies in Ab-binding assays and PBNA. Laboratory median estimated antibody concentrations are listed for samples scored overall positive.

Sample		I	*I*	J	**K**	*K*	L	M	N	O	P	P
**HPV52 Assay**	Lab Code	2v vaccinee Reference	*Relative to B*	HPV33 IS 19/290	**HPV52 IS 19/296**	*Relative to B*	HPV58 IS 19/300	Natural HPV68	HPV31 IS 20/176	HPV45 IS 20/178	2v vaccinee Reference	*Relative to B*
Ab-Binding (units/mL or titer)	7	P 55	*0.99*	N	**P 53**	*0.96*	N	N	N	N	P 54	0.97
	1	N	*nc*	N	**P 5.5**	*0.83*	N	N	N	N	N	nc
	10	P 13	*0.50*	N	**P 22**	*0.89*	N	N	N	N	P 14	0.55
	8	P 5.5	*0.38*	N	**<u>P</u>/N 10**	*0.69*	N	N	N	N	P 7.1	0.50
	6	* 6400	*3.20*	*	*** 4000**	*2.00*	*	*	*	*	* 6400	3.20
PBNA (titer)	7	N	*na*	N	**P 419**	*0.78*	N	N	N	N	N	*na*
	10	P/<u>N</u>	*na*	N	**P 1139**	*0.95*	N	N	N	N	P/<u>N</u>	*na*
	5	N	*na*	N	**P 450**	*1.00*	N	N	N	N	N	*na*
	4	<u>P</u>/N 81	*na*	N	**P 111**	*0.85*	N	N	N	N	P/<u>N</u>	*na*
	8	N	*na*	N	**P 588**	*1.12*	N	N	N	N	N	*na*
	6	P 80	*na*	N	**P 640**	*1.00*	N	<u>P</u>/N 40	N	N	P 80	*na*
	3	<u>P</u>/N 25	*na*	N	**P 311**	*1.18*	N	<u>P</u>/N 22	N	N	<u>P</u>/N 25	*na*
	11	P 63	*na*	N	**P 801**	*1.03*	N	P/<u>N</u>	N	N	P 57	*na*

Abbreviations/Notes: **P**, positive (no shading). **N**, negative (dark grey). **P/N**, both positive and negative results were obtained (light grey: <u>underline</u> indicates overall result). *****, Lab-6 cut-off not established- sample not scored. Titers are shown for Lab-6 where samples are consensus-positive across laboratories. Column in **bold type** highlights the results for the candidate International Standard for HPV52 Ab. Values in *Italics* indicate potencies relative to the candidate International Standard for HPV52 Ab (Sample B). **na**, not applicable; **nc**, not calculated. Sample and/or candidate IS scored negative.

Supplementary Table 11. HPV58 assays: Study samples scored as positive or negative for HPV antibodies in Ab-binding assays and PBNA. Laboratory median estimated antibody concentrations are listed for samples scored overall positive.

Sample		X	X	A	B	C	D	E	F	G	H	I	I	J	K	**L**	M	N	O	P	P
**HPV58 Assay**	Lab Code	FNLCR 9V Ref	*Relative to L*	HPV33 IS	HPV52 IS	Nat. HPV33	Nat. HPV52	Neg	HPV6 IS	HPV11 IS	Nat. HPV6	PHE Ref	*Relative to L*	HPV33 IS	HPV52 IS	**HPV58 IS**	Natural HPV68	HPV31 IS	HPV45 IS	PHE Ref	*Relative to L*
Ab-Binding (units/mL or titer)	7	P<br>787	*27.25*	P/N<br>3	N	P<br>4	N	N	N	P/N	N	P<br>35	1.23	P/N<br>3	N	**P<br>29**	N	N	N	P<br>33	1.14
	1	P<br>120	*80.00*	N	N	P/N	N	N	N	N	N	P<br>1	0.67	N	N	**P<br>1.5**	N	N	N	P<br>1.2	0.80
	10	P<br>941	*17.80*	N	N	N	N	N	N	N	N	P<br>13	0.25	N	N	**P<br>53**	N	N	N	P<br>14	0.26
	8	P<br>605	*46.99*	N	N	P/N<br>2	N	N	N	N	N	P<br>14	1.11	N	N	**P<br>13**	N	N	N	P<br>17	1.29
	6	*<br>48000	*24.00*	*	*	*	*	*	*	*	*	*<br>6400	3.20	*	*	***<br>2000**	*	*	*	*<br>6400	3.20
PBNA (titer)	7	P<br>5020	*14.22*	N	N	N	N	N	N	P<br>25	N	P/N	na	N	N	**P<br>353**	P<br>10	N	N	N	na
	10	P<br>17767	*12.30*	N	N	P/N	N	N	N	P<br>124	N	N	na	N	N	**P<br>1445**	P<br>108	N	N	N	na
	5	P<br>8100	*18.00*	N	N	P/N	N	N	N	N	N	N	na	N	N	**P<br>450**	N	N	N	N	na
	4	P<br>11606	*4.94*	N	N	P/N	N	N	N	N	N	P/N	na	N	N	**P<br>2349**	N	N	N	P/N	na
	8	P<br>10407	*29.07*	N	N	N	N	N	N	N	N	N	na	N	N	**P<br>358**	N	N	N	N	na
	6	P<br>5120	*4.00*	N	N	P/N<br>80	N	N	N	N	N	N	na	N	N	**P<br>1280**	P<br>40	N	N	N	na
	3	P<br>4885	*11.74*	N	N	P/N	N	N	N	P<br>27	N	N	na	N	N	**P<br>416**	P/N	N	N	N	na
	11	P<br>43098	*25.50*	N	N	P/N<br>42	N	N	N	P<br>61	N	P/N<br>43	na	N	N	**P<br>1690**	P/N<br>58	N	N	N	na

Abbreviations/Notes: **P**, positive (no shading). **N**, negative (dark grey). **P/N**, both positive and negative results were obtained (light grey: underline indicates overall result). *****, Lab-6 cut-off not established-sample not scored. Titers are shown for Lab-6 where samples are consensus-positive across laboratories. Column in **bold type** highlights the results for the candidate International Standard for HPV58 Ab. Values in *Italics* indicate potencies relative to the candidate International Standard for HPV58 Ab. **na**, not applicable.

Supplementary Table 12. Reactivity of candidate standards in PBNA and Ab-binding assays. P = positive; N = negative; P/N = both positive and negative results were obtained; underline indicates overall result across independent assays; yellow highlight indicates exception to the consensus; * = cut-off not established; - = assay not performed.

Lab	Candidate IS (Sample code)	Ab-binding assay for HPV Type								
		6	11	16	18	31	33	45	52	58
7	HPV6 Ab (F)	P	P/N	N	N	N	N	N	N	N
2		*	*	*	*	-	-	-	-	-
1		P	N	N	N	N	N	P	N	N
10		P	N	N	N	N	N	N	N	N
8		N	N	N	N	N	N	N	N	N
6		*	*	*	*	*	*	*	*	*
		PBNA for HPV Type								
		6	11	16	18	31	33	45	52	58
9		-	-	-	N	-	-	N	-	-
7		P	N	N	N	N	N	N	N	N
10		P	N	N	N	N	N	N	N	N
5		P	N	N	N	N	N	N	N	N
4		P	N	N	N	N	N	N	N	N
8		P	N	N	N	N	P/N	N	N	N
6		P	N	N	N	N	N	N	P/N	N
3		P	N	N	N	N	N	N	N	N
11		P	N	N	N	N	N	N	N	N
		Ab-binding assay for HPV Type								
		6	11	16	18	31	33	45	52	58
7	HPV11 Ab (G)	N	P	N	N	P/N	P	N	N	P/N
2		*	*	*	*	-	-	-	-	-
1		P/N	P	N	N	N	N	N	N	N
10		N	P	N	N	N	N	N	N	N
8		N	N	N	N	N	N	N	N	N
6		*	*	*	*	*	*	*	*	*
		PBNA for HPV Type								
		6	11	16	18	31	33	45	52	58
9		-	-	-	N	-	-	P/N	-	-
7		P/N	P	N	N	N	P	N	P/N	P
10		P	P	N	N	N	P	N	P	P
5		P	P	N	N	N	P	N	P/N	N
4		N	N	N	N	P/N	P	N	N	N
8		N	P	N	N	N	P	N	P	N
6		N	N	N	N	P/N	P	N	P	N
3		P	P	N	N	N	P	N	P	P
11		P	P	N	N	N	P	N	P/N	P
		Ab-binding assay for HPV Type								
		6	11	16	18	31	33	45	52	58
7	HPV31 Ab (N)	N	N	N	N	P	N	N	N	N
2		*	*	*	*	-	-	-	-	-
1		N	N	N	N	P	N	N	N	N
10		N	N	N	N	P	N	N	N	N
8		N	N	N	N	P/N	N	N	N	N
6		*	*	*	*	*	*	*	*	*

continued

Lab										
		PBNA for HPV Type								
		6	11	16	18	31	33	45	52	58
9	HPV31 Ab (N)	-	-	-	N	-	-	N	-	-
7		N	N	N	N	P	N	N	N	N
10		N	N	N	N	P	N	N	N	N
5		N	N	N	N	P	N	N	N	N
4		N	N	N	N	P	N	N	N	N
8		N	N	N	N	P/N	N	N	N	N
6		N	N	N	N	P	N	N	N	N
3		P/N	N	N	N	P	N	N	N	N
11		P	N	N	N	P	N	N	N	N
		Ab-binding assay for HPV Type								
		6	11	16	18	31	33	45	52	58
7	HPV33 Ab (dup A)	N	N	N	N	N	P	N	N	P/N
2		*	*	*	*	-	-	-	-	-
1		N	N	N	N	N	P	N	N	N
10		N	N	N	N	N	P	N	N	N
8		N	N	N	N	N	P	N	N	N
6		*	*	*	*	*	*	*	*	*
7	HPV33 Ab (dup J)	N	N	N	N	N	P	N	N	P/N
2		*	*	*	*	-	-	-	-	-
1		N	N	N	N	N	P	N	N	N
10		N	N	N	N	N	P	N	N	N
8		N	N	N	N	N	P/N	N	N	N
6		*	*	*	*	*	*	*	*	*
		PBNA for HPV Type								
		6	11	16	18	31	33	45	52	58
9	HPV33 Ab (dup A)	-	-	-	N	-	-	N	-	-
7		N	N	N	N	N	P	N	N	N
10		N	P/N	N	N	N	P	N	N	N
5		N	N	N	N	N	P	N	N	N
4		N	N	N	N	N	P	N	N	N
8		N	N	N	N	N	P	N	N	N
6		N	N	N	N	N	P	N	N	N
3		N	N	N	N	N	P	N	N	N
11		N	P/N	N	N	N	P	N	N	N
9	HPV33 Ab (dup J)	-	-	-	N	-	-	N	-	-
7		N	N	N	N	N	P	N	N	N
10		N	P/N	N	N	N	P	N	N	N
5		N	N	N	N	N	P	N	N	N
4		N	N	N	N	N	P	N	N	N
8		N	N	N	N	N	P	N	N	N
6		N	N	N	N	N	P	N	N	N
3		N	N	N	N	N	P	N	N	N
11		N	P/N	N	N	N	P	N	N	N
		Ab-binding assay for HPV Type								
		6	11	16	18	31	33	45	52	58
7	HPV45 Ab (O)	N	N	N	N	N	N	P	N	N
2		*	*	*	*	-	-	-	-	-
1		N	N	N	N	N	N	P	N	N
10		N	N	N	N	N	N	P	N	N
8		N	N	N	N	N	N	P	N	N
6		*	*	*	*	*	*	*	*	*

continued

Lab		6	11	16	18	31	33	45	52	58
		PBNA for HPV Type								
		6	11	16	18	31	33	45	52	58
9	HPV45 Ab (O)	-	-	-	N	-	-	P/N	-	-
7		N	N	N	N	N	N	P	N	N
10		N	N	N	N	N	N	P	N	N
5		N	N	N	N	N	N	P	N	N
4		N	N	N	N	N	N	N	N	N
8		N	N	N	N	N	N	P/N	N	N
6		N	N	N	N	N	N	P	N	N
3		P/N	N	N	N	N	N	P	N	N
11		P	N	N	N	N	N	P	N	N
		Ab-binding assay for HPV Type								
		6	11	16	18	31	33	45	52	58
7	HPV52 Ab (dup B)	N	P/N	N	N	N	P/N	N	P	N
2		*	*	*	*	-	-	-	-	-
1		N	N	N	N	N	N	N	P	N
10		N	N	N	N	N	N	N	P	N
8		N	N	N	N	N	N	N	P	N
6		*	*	*	*	*	*	*	*	*
7	HPV52 Ab									
	(dup K)	N	N	N	N	N	P/N	N	P	N
2		*	*	*	*	-	-	-	-	-
1		N	N	N	N	N	N	N	P	N
10		N	N	N	N	N	N	N	P	N
8		N	N	N	N	N	N	N	P/N	N
6		*	*	*	*	*	*	*	*	*
		PBNA for HPV Type								
		6	11	16	18	31	33	45	52	58
9	HPV52 Ab									
	(dup B)	-	-	-	N	-	-	N	-	-
7		N	N	N	N	N	N	N	P	N
10		N	N	N	N	N	N	N	P	N
5		N	N	N	N	N	N	N	P	N
4		N	N	N	N	N	N	N	P	N
8		N	N	N	N	N	N	N	P	N
6		N	N	N	N	N	N	N	P	N
3		P/N	N	N	N	N	N	N	P	N
11		P	N	N	N	N	N	N	P	N
9	HPV52 Ab	-			N			N	-	-
	(dup K)		-	-		-	-			
7		N	N	N	N	N	N	N	P	N
10		N	N	N	N	N	N	N	P	N
5		N	N	N	N	N	N	N	P	N
4		N	N	N	N	N	N	N	P	N
8		N	N	N	N	N	N	N	P	N
6		N	N	N	N	N	N	N	P	N
3		P/N	N	N	N	N	N	N	P	N
11		P	N	N	N	N	N	N	P	N
		Ab-binding assay for HPV Type								
		6	11	16	18	31	33	45	52	58
7	HPV58 Ab (L)	N	N	N	N	N	N	N	N	P
2		*	*	*	*	-	-	-	-	-
1		N	N	N	N	N	N	N	N	P
10		N	N	N	N	N	N	N	N	P
8		N	N	N	N	N	N	N	N	P
6		*	*	*	*	*	*	*	*	*

continued

lab		PBNA for HPV Type								
		6	11	16	18	31	33	45	52	58
	HPV58 Ab (L)	-	-	-	N	-	-	N	-	-
7		N	N	N	N	N	N	N	N	P
10		N	N	N	N	N	N	N	N	P
5		N	N	N	N	N	N	N	N	P
4		N	N	N	N	N	N	N	N	P
8		N	N	N	N	N	N	N	N	P
6		N	N	N	N	N	N	N	N	P
3		N	N	N	N	N	N	N	N	P
11		P/N	P	N	N	N	N	N	N	P

P = positive; N = negative; P/N = both positive and negative results were obtained; underline indicates overall result across independent assays; yellow highlight indicates exception to the consensus; * = cut-off not established; - = assay not performed.

**Supplementary Table 13**. External HPV reference laboratories	
Name	Laboratory
Jianhui Nie<br>Nan Xu	National Institutes for Food and Drug Control, Beijing, PR China
Joakim Dillner<br>Carina Eklund	Karolinska Institutet, Karolinska Universitessjukhuset, Huddinge, Sweden
Ligia A. Pinto<br>Troy J. Kemp	Frederick National Laboratory for Cancer Research, Frederick Maryland, USA
Elizabeth Unger<br>Gitika Panicker	Centers for Disease Control and Prevention, Chronic Viral Diseases Branch, Atlanta Georgia, USA
Four external reference laboratories undertook validation testing of the source donations prior to their selection for further development. Two reference laboratories (REF-1 and REF-2) also tested candidate materials in pre-fill mixing studies, post-fill validation and stability assessments.	

Supplementary Table 14. Summary of results from accelerated thermal degradation studies of candidate standards HPV6, 31, 33, 45, 52 and 58 tested by reference laboratory REF-1 using the PBNA method.

Candidate Standard	Time (years)	Temperature (ºC)	Lower 95% CL	**GM Potency relative -70ºC sample**	Upper 95% CL	Predicted loss per year at-20ºC
HPV6	1.14	-20	0.65	**0.96**	1.42	*
		4	0.72	**0.91**	1.16	
		20	0.76	**0.96**	1.23	
		37	0.62	**0.98**	1.54	
		45	0.39	**0.51**	0.67	
HPV31	0.46	-20	0.59	**0.93**	1.47	*
		4	0.85	**1.12**	1.47	
		20	0.84	**1.04**	1.29	
		37	0.88	**1.28**	1.87	
		45	0.43	**0.79**	1.46	
HPV33	1.64	-20	0.54	**1.18**	2.55	*
		4	0.58	**1.02**	1.77	
		20	0.89	**1.22**	1.67	
		37	0.97	**1.35**	1.87	
		45	0.20	**0.28**	0.40	
HPV45	0.46	-20	0.43	**0.80**	1.51	*
		4	0.67	**0.97**	1.39	
		20	0.39	**0.84**	1.81	
		37	0.76	**0.99**	1.29	
		45	0.42	**0.72**	1.24	
HPV52	1.64	-20	0.82	**1.02**	1.28	*
		4	0.61	**0.95**	1.48	
		20	0.77	**1.07**	1.50	
		37	0.60	**0.87**	1.25	
		45	0.34	**0.45**	0.60	
HPV58	1.14	-20	0.94	**1.46**	2.27	*
		4	0.84	**1.22**	1.79	
		20	0.77	**1.33**	2.31	
		37	1.20	**1.28**	1.36	
		45	0.39	**0.54**	0.76	

Notes: Results shown are unweighted geometric mean (GM) relative potencies calculated using laboratory-reported estimates of antibody concentration (n= 3 independent assays). A value of 1 was assigned to the -70ºC baseline sample for the purpose of the assessment. Significant losses (when compared to -70ºC) were only observed for +45 samples stored for 1.14 or 1.64 years. *Significantly poor model fit, no prediction of loss made

Supplementary Table 15. Summary of results from accelerated thermal degradation studies of candidate standards HPV6, 31, 33, 45*, 52 and 58 tested by reference laboratory REF-2 using the PBNA method.

Candidate Standard	Time (years)	Temperature (ºC)	Lower 95% confidence limit	**GM Potency relative to -70ºC sample**	Upper 95% confidence limit	Predicted loss per year at -20ºC
HPV6	1.14	-20	0.98	**1.10**	1.22	<0.001%
		4	0.55	**0.99**	1.78	
		20	0.60	**1.09**	2.00	
		37	0.77	**0.91**	1.06	
		45	0.30	**0.62**	1.27	
HPV31	0.46	-20	0.67	**1.18**	2.10	**
		4	0.70	**1.01**	1.45	
		20	0.25	**0.85**	2.88	
		37	0.65	**1.42**	3.09	
		45	0.85	**0.99**	1.15	
HPV33	1.64	-20	0.92	**1.21**	1.61	**
		4	0.80	**1.12**	1.56	
		20	0.73	**1.09**	1.63	
		37	0.70	**0.98**	1.37	
		45	0.15	**0.47**	1.51	
HPV52	1.64	-20	0.71	**1.05**	1.56	0.001%
		4	0.62	**1.21**	2.37	
		20	0.39	**1.11**	3.14	
		37	0.49	**0.83**	1.42	
		45	0.42	**0.61**	0.87	
HPV58	1.14	-20	0.41	**1.04**	2.65	**
		4	0.52	**0.91**	1.60	
		20	0.47	**1.09**	2.48	
		37	0.58	**0.95**	1.54	
		45	0.30	**0.67**	1.52	

Notes: Results shown are unweighted geometric mean (GM) relative potencies calculated using laboratory-reported estimates of antibody concentration (n= 3 or 4 independent assays). A value of 1 was assigned to the -70ºC baseline sample for the purpose of the assessment. *Estimates not obtained for HPV45 assay due to technical issues with the assay. **no model fit, no prediction of loss made.

Supplementary Table 16. Summary of results from accelerated thermal degradation studies of HPV11 candidate standard tested by reference laboratory REF-1 and REF-2 using the PBNA method and REF-1 using the Ab-binding assay.

HPV reference laboratory (assay method)	Time	Temperature (ºC)	Lower 95% confidence limit	**GM Potency relative to -20ºC sample**	Upper 95% confidence limit	Predicted loss per year at-20ºC
REF-1 (PBNA)	2 weeks	4	0.74	**0.94**	1.21	*
		20	0.57	**0.94**	1.56	
	1 month	4	0.84	**0.88**	0.92	
		20	0.67	**0.95**	1.34	
	3 months	4	0.82	**0.87**	0.92	
		20	0.69	**0.92**	1.23	
REF-2 (PBNA)	2 weeks	4	0.91	**1.02**	1.15	*
		20	0.73	**1.01**	1.40	
	1 month	4	0.72	**0.87**	1.05	
		20	0.67	**0.84**	1.05	
	3 months	4	0.91	**1.02**	1.15	
		20	0.68	**0.99**	1.43	
REF-1 (Ab-binding)	2 weeks	4	0.96	**0.98**	1.01	22.3%**
		20	0.92	**0.95**	0.99	
	1 month	4	0.95	**0.98**	1.00	
		20	0.91	**0.95**	1.00	
	3 months	4	0.85	**0.90**	0.95	
		20	0.73	**0.81**	0.90	

Notes: Results shown are unweighted geometric mean (GM) relative potencies calculated using laboratory-reported estimates of antibody concentration (n= 3 independent assays). A value of 1 was assigned to the -20ºC baseline samples for the purpose of the assessment; Samples stored at 37ºC did not fully reconstitute and were not analyzed. No significant difference between -20°C and other samples was observed. *no model fit, no prediction of loss made. ** The lack of observable realtime degradation over relatively short time spans can result in predictions of unrealistically high degradation rates using Ab-binding assays.

**Supplementary Table 17**. Summary of results from accelerated thermal degradation studies of candidate standards tested by reference laboratory REF-1 using the Ab-binding assay.						
Candidate Standard	Time (years)	Temperature (ºC)	Lower 95% confidence limit	**GM Potency relative to -70ºC sample**	Upper 95% confidence limit	Predicted loss per year at -20ºC
HPV6	1.14	-20	0.92	**0.96**	1.00	5.23%*
		4	0.87	**0.92**	0.97	
		20	0.81	**0.88**	0.94	
		37	0.81	**0.86**	0.90	
		45	0.62	**0.68**	0.75	
HPV31	0.46	-20	0.98	**1.02**	1.06	**
		4	0.89	**0.93**	0.97	
		20	0.81	**0.87**	0.94	
		37	0.84	**0.91**	0.99	
		45	0.75	**0.77**	0.80	
HPV33	1.64	-20	0.93	**1.03**	1.14	0.14%
		4	0.90	**1.01**	1.14	
		20	0.80	**0.90**	1.01	
		37	0.72	**0.76**	0.80	
		45	0.53	**0.56**	0.59	
HPV45	0.46	-20	0.95	**1.00**	1.06	**
		4	0.90	**0.93**	0.97	
		20	0.85	**0.90**	0.94	
		37	0.91	**0.99**	1.07	
		45	0.81	**0.88**	0.95	
HPV52	1.64	-20	0.96	**1.10**	1.26	0.22%
		4	0.86	**0.99**	1.15	
		20	0.75	**0.89**	1.05	
		37	0.70	**0.81**	0.94	
		45	0.55	**0.61**	0.69	
HPV58	1.14	-20	0.93	**1.02**	1.11	1.90%*
		4	0.86	**0.94**	1.02	
		20	0.77	**0.85**	0.94	
		37	0.71	**0.79**	0.87	
		45	0.61	**0.68**	0.75	

Notes: Results shown are semi-weighted geometric mean (GM) relative potencies calculated using laboratory-reported estimates of antibody concentration (n= 3 or 4 independent assays). A value of 1 was assigned to the -70ºC baseline samples for the purpose of the assessment. For all standards, no significant difference between -20ºC and -70ºC samples was observed. *The lack of observable realtime degradation over relatively short time spans can result in predictions of unrealistically high degradation rates using Ab-binding assays. **Significantly poor model fit, no prediction of loss made.

Supplementary Table 18. Summary of results from accelerated thermal degradation studies of candidate standards tested by reference laboratory REF-2 using the Ab-binding assay.

Candidate Standard	Time (years)	Temperature (ºC)	95% Lower Confidence Limit	**Potency relative to -70ºC sample**	Upper 95% confidence limit	Predicted loss per year at -20ºC
HPV6	1.14	-20	0.95	**1.00**	1.06	0.02%
		4	0.99	**1.00**	1.02	
		20	0.87	**0.95**	1.05	
		37	0.65	**0.77**	0.91	
		45	0.51	**0.56**	0.61	
HPV31	0.46	-20	0.85	**1.04**	1.28	0.00%
		4	0.91	**1.07**	1.25	
		20	0.88	**1.05**	1.25	
		37	0.81	**0.95**	1.11	
		45	0.69	**0.80**	0.93	
HPV33	1.64	-20	0.97	**1.00**	1.03	0.01%
		4	0.93	**0.97**	1.01	
		20	0.84	**0.91**	0.98	
		37	0.73	**0.76**	0.78	
		45	0.28	**0.49**	0.85	
HPV45	0.46	-20	0.92	**1.02**	1.14	0.02%
		4	1.00	**1.02**	1.04	
		20	0.74	**0.97**	1.26	
		37	0.79	**0.91**	1.06	
		45	0.77	**0.80**	0.83	
HPV52	1.64	-20	1.00	**1.03**	1.07	0.00%
		4	0.96	**1.03**	1.11	
		20	0.94	**0.97**	1.00	
		37	0.67	**0.74**	0.81	
		45	0.33	**0.47**	0.67	
HPV58	1.14	-20	0.85	**0.95**	1.06	0.02%
		4	0.83	**0.93**	1.03	
		20	0.78	**0.90**	1.05	
		37	0.71	**0.78**	0.87	
		45	0.28	**0.53**	0.97	

Notes: Results shown are unweighted geometric mean (GM) relative potencies calculated using laboratory-reported estimates of antibody concentration (n= 3 independent assays). A value of 1 was assigned to the -70ºC baseline sample for the purpose of the assessment. For all standards, no significant difference between -20ºC and -70ºC samples was observed

Supplementary Table 19. Validation of candidate International Standards for anti-HPV6, 11, 16, 18, 31, 33, 45, 52, and 58 antibody responses in Ab-binding assays by REF-1.

Assay<br>Sample ID	HPV6 (AU/mL)	HPV11 (AU/mL)	HPV16 (IU/mL)	HPV18 (IU/mL)	HPV31 (AU/mL)	HPV33 (AU/mL)	HPV45 (AU/mL)	HPV52 (AU/mL)	HPV58 (AU/mL)
HPV6 NIBSC 19/298 duplicate 1	3.2	<	<	<	<	<	<	<	<
HPV6 NIBSC 19/298 duplicate 2	3.1	<	<	<	<	<	<	<	<
HPV6 (Candidate pre-fill mixing study)	3	<	<	<	<	<	<	<	<
HPV11 NIBSC 20/174 duplicate 1	<	4.3	<	<	<	5.6	<	<	<
HPV11 NIBSC 20/174 duplicate 2	<	4.4	<	<	<	5.5	<	<	<
HPV33 NIBSC 19/290 duplicate 1	<	<	<	<	<	19.6	<	<	<
HPV33 NIBSC 19/290 duplicate 2	<	<	<	<	<	20.2	<	<	<
HPV33 (Candidate pre-fill mixing study)	<	<	<	<	<	21.5	<	<	<
HPV52 NIBSC 19/296 duplicate 1	<	<	<	<	<	<	<	24	<
HPV52 NIBSC 19/296 duplicate 2	<	<	<	<	<	<	<	22.9	<
HPV52 (Candidate pre-fill mixing study)	<	<	<	<	<	<	<	24.6	<
HPV58 NIBSC 19/300 duplicate 1	<	<	<	<	<	<	<	<	43.4
HPV58 NIBSC 19/300 duplicate 2	<	<	<	<	<	<	<	<	45.1
HPV58 (Candidate pre-fill mixing study)	<	<	<	<	<	<	<	<	46.6
Potency of pooled sample expressed as a percentage of the freeze-dried candidate	105.0%	N/A	N/A	N/A	N/A	92.6%	N/A	95.3%	95.0%

< result below cut-off, *AU* Arbitrary Units, *IU* International Units' *N/A* not applicable
Data reviewed December 2020

Supplementary Table 20. Validation of candidate International Standards for anti-HPV6, 11, 16, 18, 31, 33, 45, 52, and 58 antibody responses in Ab-binding assays by REF-2.

Assay<br>Sample ID	HPV6 (AU/mL)	HPV11 (AU/mL)	HPV16 (IU/mL)	HPV18 (IU/mL)	HPV31 (AU/mL)	HPV33 (AU/mL)	HPV45 (AU/mL)	HPV52 (AU/mL)	HPV58 (AU/mL)
HPV6 NIBSC 19/298	48.2	2.5	<	<	<	<	<	<	<
HPV6 (Candidate pre-fill mixing study)	38.3	<	<	<	<	<	<	<	<
HPV11 NIBSC 20/174	12.4	21.0	<	<	<	4.1	<	<	<
HPV33 NIBSC 19/290	<	<	<	<	<	27.5	<	<	<
HPV33 (Candidate pre-fill mixing study)	<	<	<	<	<	28.5	<	<	<
HPV52 NIBSC 19/296	<	<	<	<	<	<	<	63.9	<
HPV52 (Candidate pre-fill mixing study)	<	<	<	<	<	<	<	64.6	<
HPV58 NIBSC 19/300	<	<	<	<	<	<	<	<	32.9
HPV58 (Candidate pre-fill mixing study)	<	<	<	<	<	<	<	<	39.0
Potency of the liquid pool expressed as a percentage of the freeze-dried candidate	79.5%	N/A	N/A	N/A	N/A	103.5%	N/A	101.1%	118.4%

< result below cut-off, *AU* Arbitrary Units, *IU* International Units' *N/A* not applicable
Data reviewed December 2020

Supplementary Table 21. Evaluation of candidate HPV31 and HPV45 International Standards prior to lyophilization for anti-HPV6, 11, 16, 18, 31, 33, 45, 52, and 58 antibody responses in antibody-binding assays by REF-1.

Assay<br>Sample ID	HPV6 (AU/mL)	HPV11 (AU/mL)	HPV16 (IU/mL)	HPV18 (IU/mL)	HPV31 (AU/mL)	HPV33 (AU/mL)	HPV45 (AU/mL)	HPV52 (AU/mL)	HPV58 (AU/mL)
HPV31 Candidate pre-fill mixing study	<	<	<	<	10.9	<	<	<	<
HPV45 Candidate pre-fill mixing study (mix 1)	<	<	<	<	<	<	25.2	<	<
HPV45 Candidate pre-fill mixing study (mix 2)	<	<	<	<	<	<	18.6	<	<

< result below cut-off, *AU* Arbitrary Units, *IU* International Units.
Data reviewed October 2020.

Supplementary Table 22. Evaluation of candidate HPV31 and HPV45 International Standards prior to lyophilization for anti-HPV6, 11, 16, 18, 31, 33, 45, 52, and 58 antibody responses in pseudovirion-based neutralization assay by REF-1.

Assay<br>Sample ID	HPV6 (Titer)	HPV11 (Titer)	HPV16 (Titer)	HPV18 (Titer)	HPV31 (Titer)	HPV33 (Titer)	HPV45 (Titer)	HPV52 (Titer)	HPV58 (Titer)
HPV31 Candidate pre-fill mixing study	<	<	<	<	219	<	<	<	<
HPV45 Candidate pre-fill mixing study (mix 1)	<	<	<	<	<	<	378	<	<
HPV45 Candidate pre-fill mixing study (mix 2)	<	<	<	<	<	<	291	<	<

Abbreviations/Notes: < = result below cut-off. Data reviewed October 2020.

Supplementary Table 23. Evaluation of candidate HPV31 and HPV45 International Standards prior to lyophilization for anti-HPV6, -11, -16, -18, -31, -33, -45, -52, and -58 antibody responses in antibody-binding assays by REF-2.

Assay<br>Sample ID	HPV6 (AU/mL)	HPV11 (AU/mL)	HPV16 (IU/mL)	HPV18 (IU/mL)	HPV31 (AU/mL)	HPV33 (AU/mL)	HPV45 (AU/mL)	HPV52 (AU/mL)	HPV58 (AU/mL)
HPV31 Candidate pre-fill mixing study	<	<	<	<	34.8	<	<	<	<
HPV45 Candidate pre-fill mixing study (mix 1)	<	<	<	<	<	<	43.2	<	<
HPV45 Candidate pre-fill mixing study (mix 2)	<	<	<	<	<	<	27.5	<	<

< result below cut-off, *AU* Arbitrary Units, *IU* International Units.
Data reviewed October 2020.

<table>
<tr><td colspan="9">Supplementary Table 24. Development and production summaries for the candidate international standards.</td></tr>
<tr><td>Candidate standard</td><td>Anti-HPV6 serum</td><td>Anti-HPV11 serum</td><td>Anti-HPV31 serum</td><td>Anti-HPV33 serum</td><td>Anti-HPV45 serum</td><td colspan="2">Anti-HPV52 serum</td><td>Anti-HPV58 serum</td></tr>
<tr><td>NIBSC code</td><td>19/298</td><td>20/174</td><td>20/176</td><td>19/290</td><td>20/178</td><td colspan="2">19/296</td><td>19/300</td></tr>
<tr><td>Source material selected through initial testing by 2 HPV reference laboratories</td><td>Pool of serum (1 donor [W520816] x 1 collection) and defibrinated pooled plasma (1 donor [S120160905] x 4 collections).</td><td>Pool of serum (2 donors [W520154, W520408] x 1 collection each).</td><td>A single serum donation [01669] mixed with negative serum to reduce low level reactivity to non-target types.</td><td>Pool of serum (1 donor [W520064] x 1 collection) and defibrinated plasma (1 donor [S1920160905] x 3 collections).</td><td>A *single serum donation [W520948] mixed with negative serum to reduce low level reactivity to non-target types.</td><td colspan="2">Pool of 2 serum samples (2 donors [W520795, 1111] x 1 collection each).</td><td>Pool of 2 serum samples (2 donors [6283, W520692] x 1 collection each) and 1 defibrinated plasma (1 donor [S7520160905] x 2 collections).</td></tr>
<tr><td>Validation summary by HPV reference laboratories REF-1 and REF-2</td><td>Confirmed reactivity to HPV6. Reactivity to HPV11 was noted in 1 assay</td><td>Confirmed reactivity to HPV11. Reactivity to HPV6 and HPV33 was also observed</td><td>Monospecific for HPV31 reactivity</td><td>Monospecific for HPV33 reactivity</td><td>Monospecific for HPV45 reactivity</td><td colspan="2">Monospecific for HPV52 reactivity</td><td>Monospecific for HPV58 reactivity</td></tr>
<tr><td>Appearance</td><td>Intact cake</td><td>Dehydrated serum</td><td>Intact cake</td><td>Intact cake</td><td>Intact cake</td><td colspan="2">Intact cake</td><td>Intact cake</td></tr>
<tr><td>Bulk volume</td><td>~800 mL</td><td>320 mL</td><td>215 mL</td><td>878 mL</td><td>233 mL</td><td colspan="2">310 mL</td><td>~700 mL</td></tr>
<tr><td>No. of ampoules filled</td><td>2915</td><td>779</td><td>765</td><td>3155</td><td>877</td><td colspan="2">1037</td><td>2541</td></tr>
<tr><td>Mean fill weight</td><td>0.2655 g</td><td>0.2669 g</td><td>0.2661 g</td><td>0.2651 g</td><td>0.2665 g</td><td colspan="2">0.2655 g</td><td>0.2651</td></tr>
<tr><td>CV of fill weight</td><td>0.9728% (n=114)</td><td>1.2 g (n=31)</td><td>1.09% (n=36)</td><td>0.9442% (n=148)</td><td>1.6724% (n=730)</td><td colspan="2">1.4243% (n=92)</td><td>0.83% (n=96)</td></tr>
<tr><td>Mean dry weight</td><td>0.02080 g</td><td>0.02075 g</td><td>0.02144 g</td><td>0.01928 g</td><td>0.02304 g</td><td colspan="2">0.02283 g</td><td>0.02176 g</td></tr>
<tr><td>CV of dry weight</td><td>3.19% (n=6)</td><td>2.05% (n=6)</td><td>1.12% (n=6)</td><td>0.74% (n=6)</td><td>1.64% (n=6)</td><td colspan="2">0.60% (n=6)</td><td>1.73% (n=6)</td></tr>
<tr><td>Mean residual moisture</td><td>0.59%</td><td>Not determined</td><td>0.45%</td><td>3.19%</td><td>0.26%</td><td>1.74%</td><td>1.04%</td><td>0.63%</td></tr>
<tr><td>CV of residual moisture</td><td>20.74% (n=12)</td><td>Not determined</td><td>15.82% (n=12)</td><td>20.45% (n=12)</td><td>14.20% (n=12)</td><td>140.03% (n=12)</td><td>21.92% (n=11)</td><td>20.32% (n=12)</td></tr>
<tr><td>Mean oxygen content</td><td>0.42%</td><td>12.37%</td><td>0.38%</td><td>0.56%</td><td>0.58%</td><td colspan="2">0.53%</td><td>0.39%</td></tr>
<tr><td>CV of oxygen content</td><td>32.25% (n=12)</td><td>17.74% (n=12)</td><td>26.71% (n=12)</td><td>9.13% (n=12)</td><td>24.24% (n=12)</td><td colspan="2">20.36% (n=12)</td><td>46.78% (n=12)</td></tr>
</table>
